# Physics-Informed Cascaded Learning for Predicting and Optimizing Geometry and Quality in Laser Cladding Repair of Carburized Gear Steel

**DOI:** 10.3390/ma19173793

**Published:** 2026-09-06

**Authors:** Yingjie Xu, Peng Zheng, Zhongming Liu, Linfan Du, Shidang Yan, Miaomiao Xie, Zhanqi Gao, Yangyang Luo

**Affiliations:** 1 School of Mechanical and Power Engineering, Zhengzhou University, Zhengzhou 450001, China; xuyjzrime@126.com (Y.X.);; 2 ZRIME Gearing Technology Co., Ltd., Zhengzhou 450001, China; zmliu2001@126.com (Z.L.);; 3 China Academy of Machinery Zhengzhou Research Institute of Mechanical Engineering Co., Ltd., Zhengzhou 450001, China

**Keywords:** laser cladding, carburized and quenched gear steel, process–geometry–quality coupling, physics-informed machine learning, cascaded multi-learner, multi-objective optimization

## Abstract

Laser cladding is promising for use in repairing carburized gear steels, but parameter selection is challenging when clad geometry, substrate thermal disturbance, and morphological quality must be considered. Fifteen tracks of NHT.22.A01 iron-based powder were deposited on carburized-quenched 18CrNiMo7-6 steel with varying laser powers, scanning speeds, and powder feed rates. A physics-informed cascaded-learning framework predicted track width, height, Ac_1_-boundary depth, and quality. Leave-one-out out-of-fold width predictions were transferred to height and depth models to prevent target leakage. An auxiliary continuous quality index enabled the bi-objective optimization of quality and high-hardness layer depth, while multi-indicator process maps with local sensitivity analysis supported rapid parameter adjustment. Leave-one-out (R^2^) values for three geometric responses ranged from 0.934 to 0.968, and the maximum relative error at an unseen boundary condition was 4.3%. The fitted HAZ-depth/track width scaling coefficient (0.211) lay within the Rosenthal theoretical range (0.15–0.25), confirming the physical consistency of the cascade relationship. The optimization revealed a trade-off between quality and high-hardness layer depth; maximizing predicted high-hardness layer depth under $\left(Q_{cont}\geq 4.5\right)$ selected a condition already represented in the training set. Repeatability was supported by an independent batch replicate. By integrating leakage-controlled cascading, physically interpretable validation, constrained optimization, and sensitivity-resolved process mapping, the framework provides a transparent and practically applicable approach to small-sample laser cladding process design.

## 1. Introduction

Laser cladding (LC) stands as a pivotal surface modification and restoration technique for high-value components within advanced manufacturing. Characterized by high metallurgical bonding, low dilution rate, and a narrow heat-affected zone (HAZ), LC effectively enhances the wear, corrosion and fatigue resistance of structural parts [1,2]. For the remanufacturing and repair of high-value transmission components such as carburized-quenched steel gears and bearings, LC enables material replenishment and property restoration with limited thermal disturbance to the substrate [3]. Nevertheless, the consistent industrial realization of these merits strongly relies on the coordinated regulation of energy input, melt pool dynamics, a solidified microstructure and thermal cycling trajectories via tunable processing parameters [4,5]. Particularly for on-site repair and low-volume remanufacturing workflows, the core challenge extends far beyond viable deposition. Instead, researchers must ensure robust, predictable mapping between processing parameters and clad track geometry (track width *w*_1_ and height *h*) under constrained experimental budgets and inherent process variability. Furthermore, reusable processing windows were established to screen single- and multi-channel overlapping schemes that meet predefined quality thresholds [6]. Moreover, process parameter mappings derived from single-pass cladding experiments can serve as preliminary screening benchmarks for subsequent multi-pass and multi-layer cladding processes [7,8]. However, thermally induced microstructural evolution and property gradients within the HAZ severely degrade component service reliability [9]. For thermally sensitive components including gears, gradient hardening layer characteristics and corresponding property degradation induced by thermal history fluctuations demand rigorous attention [10,11]. Recent investigations confirm that characterizing the thermal disturbance boundaries of hardened layers via the Ac_1_ isotherm carries clear physical significance, alongside robust engineering feasibility for numerical modeling and experimental validation [12,13]. Accordingly, under the condition of limited data, developing a coupled predictive framework for single-track width (*w*_1_), height (*h*), Ac_1_-boundary depth (*d*), and overall quality is critical for reliable process window screening and the industrial deployment of laser cladding on carburized-quenched steel.

Extensive prior literature has laid a robust theoretical and experimental foundation for process–geometry–quality modeling and process window screening. Existing approaches fall into three main categories: physics-based mechanistic modeling, empirical surrogate correlations, and data-driven prediction. The classic moving heat-source Rosenthal model establishes a fundamental framework describing scaling laws linking temperature fields to laser power and scanning speed [14]. For the analytical modeling of single-track clad geometry, Lalas et al. constructed a geometry prediction model accounting for surface tension effects [15]. El Cheikh et al. extended this framework by incorporating how powder spatial distribution within the jet alters clad track formation [16]. From an industrial perspective, linear energy density (LED=P/v) and volumetric energy density (VED) serve as widely adopted empirical surrogates to correlate clad geometry and thermal evolution [17]. These metrics are further applied to preliminary evaluations of macroscale metallurgical quality metrics, including porosity and crack susceptibility [18]. Nevertheless, accumulating evidence demonstrates that standalone energy density metrics fail to capture heterogeneous laser absorption and powder deposition behavior, while exhibiting poor transferability across different equipment and material systems. This limitation motivates the development of mechanism-oriented feature descriptors or refined energy calculation models [19].

Data-driven modeling has been increasingly employed in laser cladding to capture complex process–response relationships. Early studies primarily used conventional linear regression and the response surface methodology (RSM) to quantify the effects of laser power *p*, scanning speed *v*, and powder feed rate *F* on track morphology [20]. Although these approaches provide transparent process–response relationships, ordinary least-squares estimates can become unstable when the predictors are strongly correlated, particularly when the number of experiments is limited.

Among the regularized linear estimators, Ridge regression is particularly relevant to small datasets containing correlated predictors. Hoerl and Kennard introduced Ridge regression for nonorthogonal designs by adding an L_2_ penalty to the least-squares objective [21,22]. The resulting coefficient shrinkage deliberately introduces a small amount of bias, but can substantially reduce estimation variance and prediction error. Subsequent work established cross-validation-based procedures for selecting the regularization parameter, making the degree of shrinkage data-dependent rather than arbitrary [23]. Ridge regression retains all predictors and tends to stabilize the joint contribution of correlated variables; however, it does not perform exact variable selection, and its estimates depend on appropriate feature scaling [24]. Beyond regularized linear modeling, genetic-algorithm-assisted back-propagation neural networks [25] and conventional artificial neural networks [26,27] have been employed to improve nonlinear predictions of single-track geometry. Multi-output support vector regression [28] and least-squares support vector regression [29] have also been used for geometric prediction and process-parameter optimization. However, these studies have primarily focused on responses such as track width, height, penetration depth, and dilution, which do not directly quantify thermal alteration within the carburized case. Dychtoń et al. [30] showed that the boundary associated with the Ac_1_ temperature delineates the region in which hardness changes abruptly. Despite its physical relevance to thermally affected carburized layers, the Ac_1_-boundary depth has rarely been incorporated as a response in laser-cladding surrogate models.

Machine learning studies have also addressed quality-related responses, including microhardness [31] and crack density [32], while knowledge-transfer strategies have linked information across related laser-processing prediction tasks [33]. In a sequential modeling framework, however, using in-sample upstream predictions as downstream inputs can introduce information leakage and produce overly optimistic performance estimates. Cross-validation-generated out-of-fold predictions therefore provide a more appropriate basis for transferring information between modeling stages [34,35]. Interpretability and uncertainty quantification are also important for process development: SHAP has been used to quantify the contributions of process and material descriptors [36], whereas GPR has been applied to cladding track geometry because it provides both response predictions and model-derived uncertainty estimates [37]. More recently, GP prediction has been integrated with SHAP-based interpretation to support laser-cladding process optimization [38]. Nevertheless, the leakage-controlled integration of regularized regression, nonlinear learning, hierarchical geometry, physical interpretation, and uncertainty-aware optimization remains insufficiently investigated for small laser-cladding datasets.

To address these challenges, this study develops a leakage-controlled, physics-informed cascaded-learning framework for the joint prediction of track morphology (width, height, and Ac_1_-boundary HAZ depth) and quality. The framework adopts a multi-learner ensemble approach to capture both linear and non-linear process–property interactions, while preventing target leakage through cascaded transfer. An auxiliary quality index is constructed to support multi-objective optimization, balancing dimensional accuracy with morphological quality. Finally, the framework’s robustness is validated through separate-batch replicates and unseen boundary conditions, providing a reliable basis for rapid parameter adjustment within the design space.

## 2. Materials and Methods

### 2.1. Materials, Experimental Procedure, and Characterization

**Figure 1:**
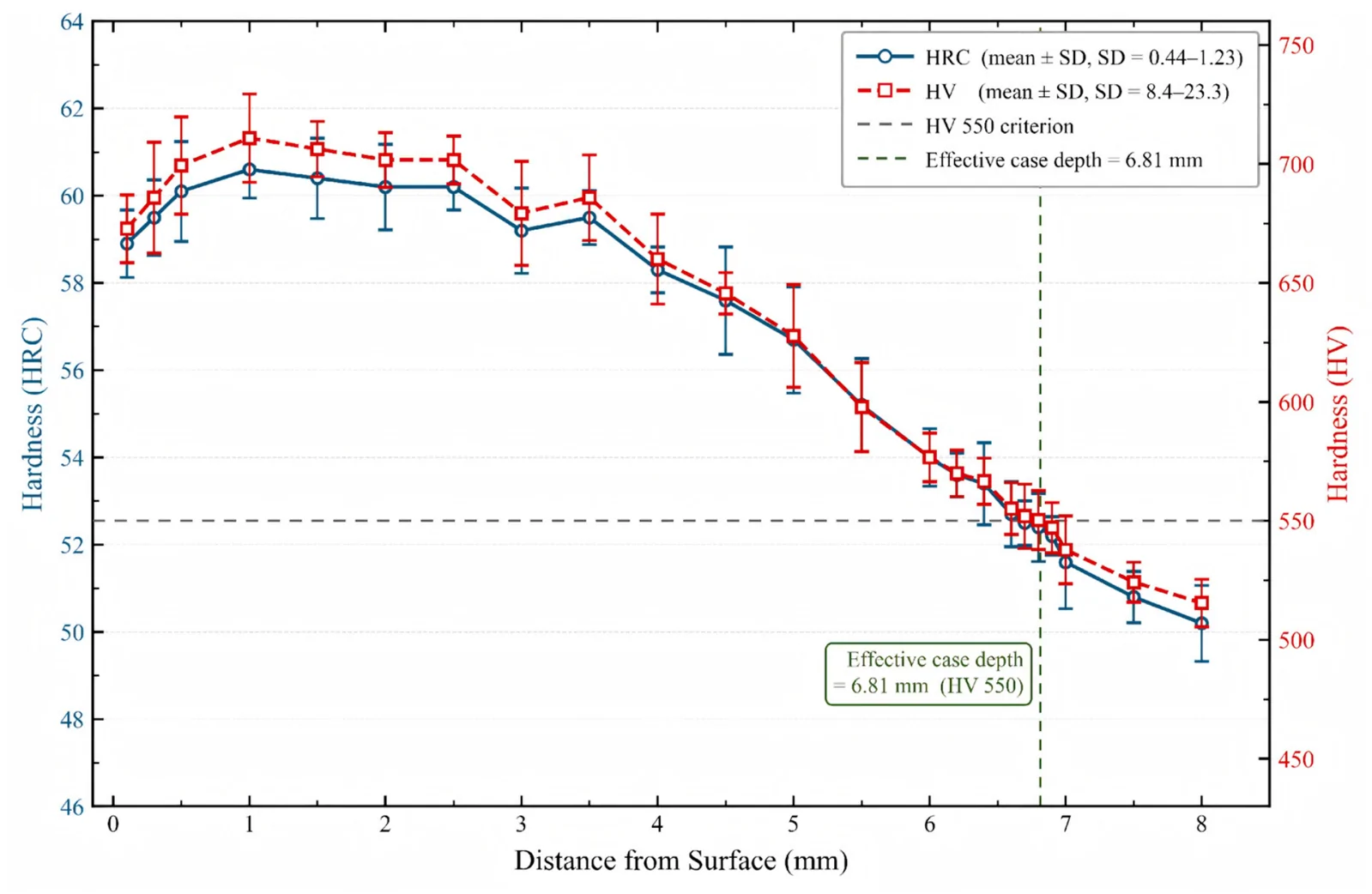
Hardness distribution profiles of the 18CrNiMo7-6 substrate.

Single-track, single-layer laser cladding experiments were performed on carburized-quenched 18CrNiMo7-6 steel substrates in this work. Prior to cladding deposition, the chemical composition and depth-dependent hardness gradient of the substrate were characterized, with corresponding results summarized in Table 1 and Figure 1, respectively.

Gas-atomized iron-based alloy powder NHT.22.A01 supplied by Höganäs was selected as the cladding feedstock, with particle sizes ranging from 20 to 65 μm. The manufacturer-provided elemental composition of this powder is listed in Table 2. A high-power Laserline LDF-6000 diode laser system was utilized for single-track, single-layer deposition, integrated with a GTV 800 processing head and coaxial powder-feeding nozzle. The laser operated at a wavelength of 975 nm with a fixed spot diameter of 3 mm. Laser power *P* (W), scanning speed *v* (mm/s) and powder feed rate *F* (mg/s) were defined as controllable processing variables. A total of 15 distinct parameter combinations were tested (see the first four columns in Table 3).

**Figure 2:**
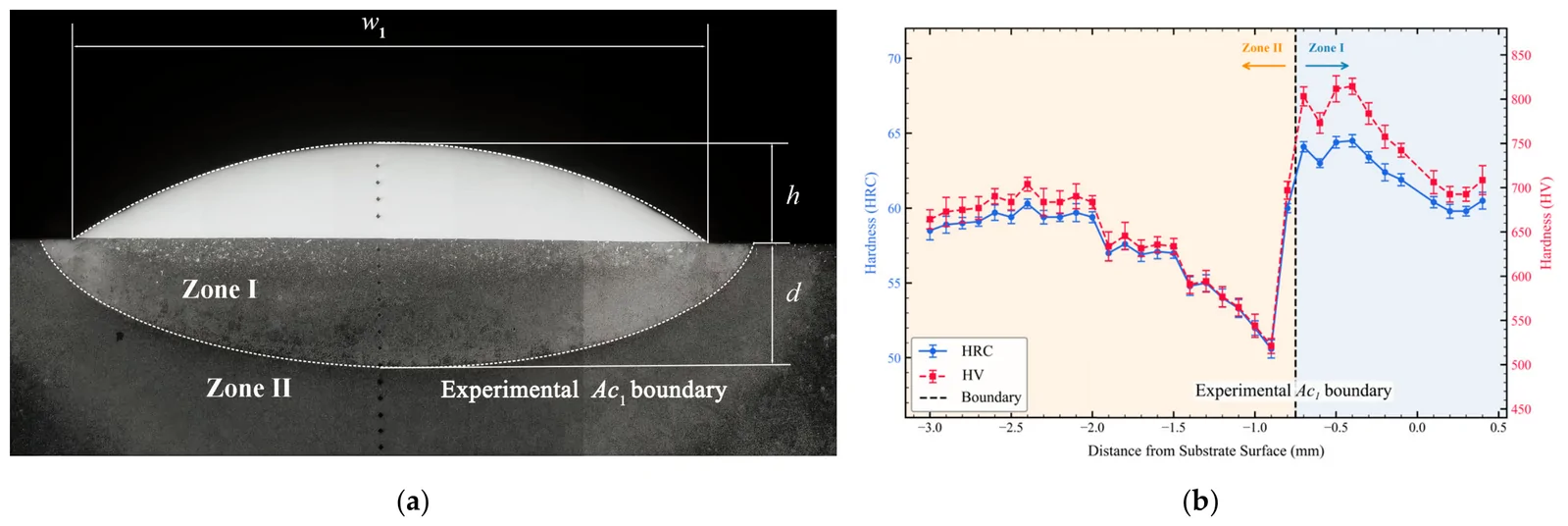
Comparison of the boundary microstructure and hardness gradient of Ac1: (**a**) microstructure; (**b**) hardness gradient.

After cladding fabrication, cross-sectional specimens were excised perpendicularly to the scanning direction from the structurally stable region of each single clad track. Subsequently, metallographic specimens with a dimension of 10 mm × 10 mm were prepared via standard grinding and polishing procedures. The specimens were etched for 10 s using a 4% volume fraction nital solution and characterized via a Nikon MA200 optical microscope at 50× magnification. As displayed in Figure 2a, chemical etching generated a distinct, sharp optical boundary between the bright quenched martensite (Zone I) and dark tempered martensite (Zone II).

To establish a precise correlation between microstructural evolution and mechanical performance, microhardness depth profiles were measured along the central line of each cross-section using an HXD-1000 microhardness tester. Each measurement was repeated across three independent cross-sectional specimens (*n* = 3) to ensure statistical significance. The results are reported as the arithmetic mean ± sample standard deviation, which are represented by the error bars in Figure 2b. This approach accounts for local material variability and ensures the reliability of the observed hardness gradients. The original substrate surface was defined as the coordinate origin, with the direction toward the cladding layer set as the positive axis. The test results in Figure 2b show a precise correspondence between the inflection point of the hardness gradient and the microstructural boundary. This interface defines the lower limit of the high-hardness region, and in this study the distance from this interface to the original substrate surface is defined as the *Ac*_1_-boundary depth (denoted *d*). The depth value d directly represents the depth of the hard layer after laser cladding on carburized and quenched steel, and serves as a key criterion for delineating mechanical property zones. Wang et al. [10] reported that when the surface hardness of carburized and quenched gears exceeds 700 HV, the location of maximum local fatigue-failure risk shifts from near the surface to the transition layer (the hardness/strength softening zone), substantially increasing spalling risk. The greater the depth of the hardened layer, the more likely it is that the region of high surface shear stress remains above the softening zone interface, thereby reducing spalling risk [39].

**Figure A1:**
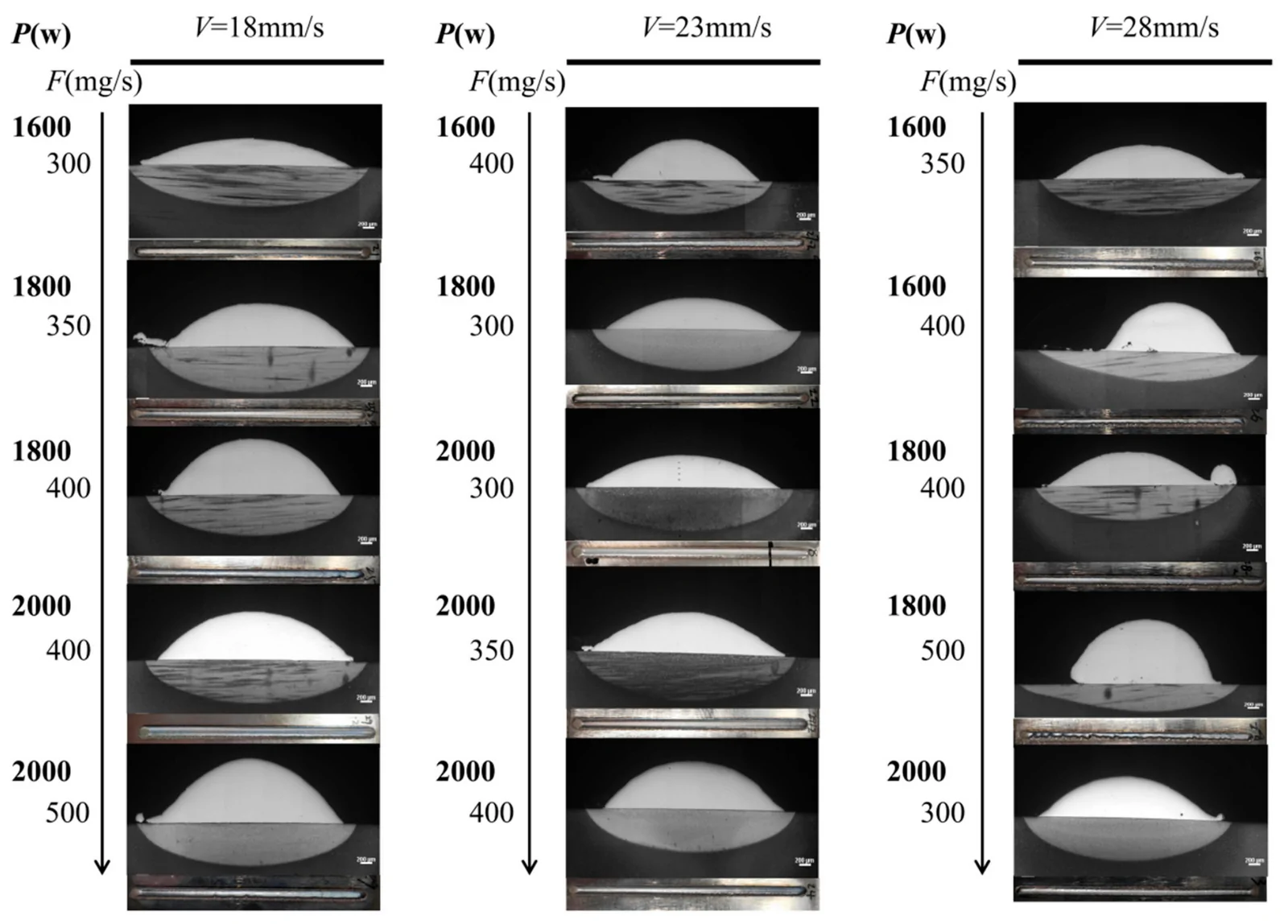
Cross-sectional optical micrographs of the clad tracks produced under the 15 experimental conditions. The scale bar in each panel was generated from the corresponding pixel-to-length calibration.

Subsequently, ImageJ 1.54g software was utilized for the scale calibration and horizontal correction of metallographic images. Curve fitting was performed on clad track profiles and isothermal boundaries to precisely extract three core geometric parameters (illustrated in Figure 2a): track width *w*_1_ (maximum cross-sectional width of the clad track, mm), track height *h* (maximum height above the substrate surface, mm), and *d* (mm). To guarantee the statistical reliability of experimental data, each geometric parameter was measured at three distinct positions along the track length, and average values were calculated. Figure A1 in Appendix A presents the raw metallographic micrographs of all specimens.

Single-track morphology was evaluated using a predefined five-level ordinal rubric, (Q∈1,…,5), combining macroscopic track appearance with metallographic cross-sectional characteristics (Table 4). The criteria were established before model fitting and covered track continuity, balling or humping, powder adhesion, geometric uniformity, cross-sectional symmetry, and toe wetting.

Top-view and cross-sectional micrographs were anonymized and independently scored by two domain experts in randomized order. Both assessors were blinded to the processing parameters and model predictions. The assessment was repeated after a 10-day washout period. Inter- and intra-observer agreement was evaluated using quadratic-weighted Cohen’s kappa, exact-grade agreement, and within-one-grade agreement. Discrepant ratings were resolved through joint review, and the resulting consensus grade, $Q_{dis,i}$, was used as the observed quality label for specimen (i). The consensus grades and agreement statistics are reported in Section 3.1.

For weighted fitting of the auxiliary continuous-quality response, a quality-derived sample weight $s_{i}$ was assigned from $Q_{dis,i}$;

(1) $$\begin{array}{c} s_{i}=\left\{\begin{array}{ll} 0.100, & Q_{dis,i}\in \left\{1,2\right\}\\ 0.220\times e^{\left(Q_{dis,i}-3\right)}, & Q_{dis,i}\in \left\{3,4,5\right\} \end{array}\right. \end{array}$$

These weights controlled the contribution of individual specimens to the regression loss without modifying the measured responses or descriptor values. Quality-based weighting was used in the weighted learners for width and height prediction and in constructing the auxiliary continuous quality response, whereas the depth model was trained with equal sample weights.

### 2.2. Physics-Informed Cascaded Framework for Geometry Prediction

#### 2.2.1. Physics-Informed Feature Engineering and Cascaded Model Development

Laser power *P* (W), scanning speed *v* (mm/s) and powder feed rate *F* (mg/s) were normalized into dimensionless forms via division by their respective reference values, as follows:

(2) $$\tilde{P}=\frac{P}{P_{ref}},\;\tilde{v}=\frac{v}{v_{ref}},\;\tilde{F}=\frac{F}{F_{ref}}$$

All reference values correspond to the mean values of the experimental dataset; $P_{ref}$ = 1826.67 W, $v_{ref}$ = 23.00 mm/s, $F_{ref}$ = 376.67 mg/s. Linear energy density (LED) and volumetric energy density (VED) are defined as composite physical descriptors, as follows:

(3) $$LED=\frac{\tilde{P}}{\tilde{v}},\;VED=\frac{\tilde{P}}{\tilde{v}\tilde{F}}$$

where the units of LED and VED are J·mm^−1^ and J·mg^−1^, respectively.

To systematically expand the predictive feature space while capturing non-linear process mechanics, an initial library of 39 physics-informed canonical descriptors across four categories (C1–C4) was constructed (Table A1). To prevent overfitting under the small-sample regime (*N* = 15), a rigorous two-stage dimensionality reduction protocol was applied. First, pairwise Pearson correlation analysis was conducted to eliminate severely collinear candidates (∣*r*∣ > 0.92), drastically shrinking the candidate space from *n*^raw^ (30–40) down to *n*^col^ (11–14) for each target (Table A2).

Based on mass conservation and Rosenthal analytical thermal constraints, the three-dimensional geometry was decomposed into a hierarchical dependency chain—width (*w*_1_) → height (*h*) → depth (*d*). Specifically, *w*_1_ was modeled using primary process parameters; subsequently, its cross-validated prediction was introduced as a higher-order C4 cascade descriptor into the candidate pools for *h* and *d*. Crucially, to prevent target leakage and overly optimistic evaluations in small-sample modeling, only out-of-fold leave-one-out (LOO) predictions were passed along the cascade chain, ensuring strictly independent training at each step.

For each geometric target, three complementary base learners—Ridge regression [21], GPR [40], and ElasticNet [41]—were trained in parallel. Non-negative least squares (NNLS) [42] was implemented on the out-of-fold LOO predictions of the base learners to solve for non-negative ensemble weights—

(4) $$\hat{y}=w_{WRR}\;{\hat{y}}_{WRR}+w_{GPR}\;{\hat{y}}_{GPR}+w_{EN}\;{\hat{y}}_{EN},w_{i}\geq 0,\sum_{i=1}^{3}w_{i}=1$$

Here, $\hat{y}$ denotes the final ensemble prediction, ${\hat{y}}_{i}$ is the out-of-fold prediction generated by base learner i, and $w_{i}$ is its corresponding non-negative ensemble coefficient, where i∈(WRR,GPR,EN). The coefficients $w_{i}$ were estimated by applying non-negative least squares (NNLS) to the out-of-fold LOO predictions of the three base learners, and were subsequently normalized such that $\left(w_{i}\geq 0\right)$ and $\left(\sum_{i=1}^{3}w_{i}=1\right)$. Ridge regression provides $L_{2}$-regularized linear estimation, ElasticNet combines $L_{1}$ and $L_{2}$ regularization, and GPR represents nonlinear response behavior. Where an ensemble was retained, NNLS estimated non-negative coefficients from the out-of-fold predictions of the retained learners. The coefficients were normalized to sum to one, producing a convex combination that avoids cancellation due to negative weights and improves extrapolation stability.

The ensemble LOO-R^2^ was adopted as the objective function to determine the optimal sparse feature subsets (*n*^opt^). As summarized in Table A2, this workflow compressed the final active input dimensions to just 3, 4, and 3 features for *w*_1_, *h*, and *d*, respectively, maintaining a conservative sample-to-feature ratio (>3.5:1).

#### 2.2.2. Model Interpretation and Statistical Evaluation

Following collinearity screening and sparse subset selection, Shapley Additive Explanations (SHAP) were used to interpret the final geometric models [43]. SHAP decomposes a prediction into a reference value and the additive contributions of individual predictors,

(5) $$\begin{array}{c} {\hat{y}}_{i}=\phi_{0}+\sum_{j=1}^{p}\phi_{ij} \end{array}$$

where $\phi_{0}$ is the expected prediction over the background data and $\phi_{ij}$ is the contribution of feature *j* to observation *i*. Because the final predictors included heterogeneous learners and their retained NNLS combinations, model-agnostic KernelSHAP was applied to each complete prediction function.

The 15 standardized experimental observations were used as the background distribution. As only three, four, and three predictors were retained for *w*_1_, *h*, and *d*, respectively, all feature coalitions were evaluated. Global importance was calculated as $\begin{array}{c} I_{j}=n^{-1}\sum_{i=1}^{n}|\phi_{ij}| \end{array}$, while signed SHAP values were used to examine the direction of individual effects. SHAP was applied only after model selection and was not used for feature screening or performance evaluation.

Predictive performance was calculated from the pooled out-of-fold predictions generated by LOOCV. The coefficient of determination ($R_{LOO}^{2}$), root-mean-square error ($RMSE_{LOO}$), and mean absolute error ($MAE_{LOO}$) were defined as

(6) $$\begin{array}{c} R_{LOO}^{2}=1-\frac{\sum_{i=1}^{n}(y_{i}-{\hat{y}}_{i,LOO}{)}^{2}}{\sum_{i=1}^{n}(y_{i}-\hat{y}{)}^{2}} \end{array}$$

(7) $$\begin{array}{c} RMSE_{LOO}=\sqrt{\frac{1}{n}\sum_{i=1}^{n}(y_{i}-{\hat{y}}_{i,LOO}{)}^{2}} \end{array}$$

(8) $$\begin{array}{c} MAE_{LOO}=\frac{1}{n}\sum_{i=1}^{n}|y_{i}-{\hat{y}}_{i,LOO}| \end{array}$$

RMSE was used as the primary dimensional error because it retains the physical unit of the response and assigns greater influence to comparatively large prediction errors, which are important in process window screening [44]. MAE was calculated as a complementary metric to describe the typical absolute prediction error without disproportionately weighting extreme residuals. MSE was not reported separately because it contains the same squared-error information as RMSE but is expressed in squared physical units (mm^2^). MAPE was not used for cross-response comparisons because percentage errors can be disproportionately amplified for responses with small denominators, particularly the Ac1-boundary depth (d) [45]. The Shapiro–Wilk test was applied to the pooled LOO residuals as a supplementary distributional diagnostic [46], using a significance level of 0.05. Because only 15 observations were available and the LOO residuals were obtained from overlapping training sets, the resulting *p*-values were interpreted descriptively and were not treated as formal proof of residual normality.

#### 2.2.3. Baseline Models and Comparative Evaluation Protocol

To benchmark the predictive capability of the physics-cascaded framework, two reference models were evaluated using the same 15 experimental conditions and the same leave-one-out cross-validation (LOOCV) splits. The first reference model, denoted as Direct LED/VED, was a Ridge regression model that predicted each geometric response directly from the linear energy density (LED) and volumetric energy density (VED). The second reference model, denoted as Compact RSM, was a Ridge-regularized full quadratic response-surface model constructed from the original process parameters, with the input terms $\begin{array}{c} \tilde{P},\;\tilde{v},\;\tilde{F},\;\tilde{P^{2}},\;\tilde{v^{2}},\;\tilde{F^{2}},\;\tilde{P}\tilde{v},\;\tilde{P}\tilde{F},\;\tilde{v}\tilde{F}. \end{array}$ Separate models were established for *w*_1_, *h*, and *d*. All three models were evaluated using identical LOO partitions and the same performance metrics, including R^2^ and RMSE, to provide a consistent comparison.

### 2.3. Quality Prediction Based on Continuous Index Transformation

#### 2.3.1. Construction and Prediction of the Auxiliary Continuous Quality Response

To enable gradient-based multi-objective optimization, a continuous auxiliary response, *Q*cont, was formulated. It should be emphasized that *Q*cont does not replace the observed ordinal scores, but serves as a smooth manifold interpolating across the quality landscape via process-derived dimensionless variables,

(9) $$X_{cont}=\left[\tilde{P},\;\tilde{v},\;\tilde{F},\;LED,\;and\;VED\right]$$

A sample-weighted LOO-Ridge regression was employed for continuous quality prediction. To amplify the contribution of high-score samples (*Q* ≥ 4), a weight factor w=8.0 was assigned (baseline weight = 1.0). The Ridge regularization coefficient was automatically selected via LOO cross-validation over α∈ [10^−3^, 10^4^]. The resulting $Q_{cont}$ was clipped to [1.0, 5.0], achieving a Pearson correlation of *r* = 0.66 with the consensus ordinal scores (*Q*_dis_). This outcome confirms a moderate qualitative correspondence rather than direct numerical equivalence between the surrogate continuous metric and the discrete ratings.

This moderate level of agreement is physically and statistically consistent. The continuous model accurately reflects primary macroscopic trends—such as aspect ratio and the wetting transition—while regularizing localized stochastic features (e.g., microscopic unmolten powder adhesion and subtle surface ripples) that expert visual inspection can observe. Furthermore, given the modest sample size (*n* = 15), this continuous metric should be interpreted cautiously as an auxiliary optimization landscape rather than an absolute deterministic rating.

#### 2.3.2. Geometry-Mediated Quality Mapping

A mapping from process parameters to continuous quality indices was established, with the geometric parameters defined in Section 2.2 serving as intermediate representations.

To account for the propagation of upstream geometry prediction errors through the cascaded pipeline, a residual-calibrated data augmentation strategy was developed to improve the robustness of the second-stage quality model to input perturbations. The residual distribution of the first-stage training predictions was estimated and used as a proxy for upstream predictive uncertainty. The second-stage input features derived from the measured geometries were then perturbed accordingly.

Prediction residuals were first calculated on the training set of the first-stage geometric model (*w*_1_, *h* and *d*), and the standard deviation of residuals for each geometric variable was estimated separately. These values quantify the inherent prediction error scale of the upstream model, with results listed as follows: σ*w*1 = 0.040 mm, σ*d* = 0.014 mm, and σ*h* = 0.038 mm.

During training, residual-calibrated perturbations were injected into the measured geometric inputs, with the perturbation magnitude determined by the corresponding residual standard deviation Equation (10).

(10) $${w_{1}}^{\prime }=w_{1}+N\left(0,\sigma_{w_{1}}^{2}\right),\;d^{\prime }=d+N\left(0,\sigma_{d}^{2}\right),\;h^{\prime }=h+N\left(0,\sigma_{h}^{2}\right)$$

During inference and optimization, no explicit noise was added to the inputs. Instead, the point predictions (${\hat{w}}_{1}$, $\;\hat{h}$ and $\hat{d}$) of the first-stage geometric models were transferred directly to the second stage. This “perturbation during training” and “point prediction during inference” strategy improved robustness to upstream error propagation without introducing inference time randomness, and more closely represented practical deployment conditions.

A candidate feature pool for quality prediction was constructed, comprising a geometric feature set {${w_{1}}^{\prime },d^{\prime },h^{\prime },d^{\prime }+h^{\prime },d^{\prime }/h^{\prime },{w_{1}}^{\prime }/d^{\prime },{w_{1}}^{\prime }/h^{\prime }$} and a normalized process–geometric interaction feature set {$\tilde{p}\cdot \tilde{{w_{1}}^{\prime }},\tilde{v}\cdot \tilde{{w_{1}}^{\prime }},\tilde{F}\cdot \tilde{{w_{1}}^{\prime }},LED.\tilde{{w_{1}}^{\prime }},VED\cdot \tilde{{w_{1}}^{\prime }}$}.

Nested cross-validation was adopted to achieve leakage-controlled feature selection and performance evaluation under the small-sample condition. The outer loop used LOOCV (*n* = 15), holding out one sample per split as the test case; all feature-selection steps were performed only on the remaining (*n* − 1) training samples. Features were ranked by their Pearson correlation with the continuous quality index computed on the outer training set. Ranked subsets containing no more than two features were then evaluated, and the optimal subset was selected by the five-fold cross-validation of Ridge regression within the outer training set. Finally, the model was refit on the outer training set using the selected features, and used to predict the held-out sample. This design prevents information leakage and provides a more reliable estimate of generalization performance.

The optimal feature set for $Q_{cont}$ was determined as ($d^{\prime }$, $\tilde{p}\cdot \tilde{{w_{1}}^{\prime }}$). Based on this subset, both a Ridge model and a GPR model were trained for continuous quality prediction; the Ridge model serves as a stable baseline for point prediction, while the GPR model further provides prediction means and uncertainty estimates.

#### 2.3.3. Multi-Objective Optimization via Weighted Pareto Differential Evolution

(1) Objective function formulation

Under geometric feasibility constraints, the predicted quality $Q_{cont}$ and total hardening depth (*d + h)* were maximized simultaneously. A weighted scalarization method was adopted to explore the trade-off frontier,

(11) $$maximize\;S\left(P,v,F;w_{Q}\right)=w_{Q}.{\tilde{Q}}_{cont}+\left(1-w_{Q}\right)\cdot \tilde{\left(d+h\right)}-0.05\cdot \sigma_{Qcon}-\Psi_{pen}\;$$

where ${\tilde{Q}}_{cont}$ and $\tilde{d+h}$ denote the normalized objectives over the feasible design space, $\sigma_{Qcon}$ is the GPR prediction standard deviation (penalizing high-uncertainty regions), and $\Psi_{pen}$ is a quadratic penalty term enforcing hard constraints,

(12) $$\Psi_{pen}=\sum_{c\in \{w_{1},d,h,d+h\}}\max{\left(0,c_{\min}-c\right)}^{2}+\max{\left(0,c-c_{\max}\right)}^{2}$$

The feasibility boundaries were defined as *w*_1_ ≥ 2.5 mm, *d* ≥ 0.4 mm, *h* ≥ 0.3 mm, and 0.7 mm ≤ *d + h* ≤ 2.5 mm, and the quality weight was $w_{Q}$∈ [0.3, 1].

(2) Adaptive weight sampling and Pareto frontier extraction

To characterize the trade-off landscape, a piecewise adaptive-weight scan was performed. The interval [0.3, 1.0] was first sampled using Δ = 0.10 to identify the region of rapid objective trade-off. This region was subsequently sampled using Δ = 0.01 to locate the Pareto knee more precisely.

For each weight $w_{Q}$, differential evolution (DE) was run independently for optimization, with a population size of 18 and 400 iterations. The search boundaries were set as *P* ∈ [1600, 2000] W, *v* ∈ [18, 28] mm/s, and *F* ∈ [300, 500] mg/s. The weighted objective function $S\left(P,v,F;w_{Q}\right)$ in Equation (7) was maximized, and candidate solutions were Pareto-ranked to extract the Pareto frontier (the set of non-dominated solutions) [47]. The final recommended solution must satisfy E[*Q*_cont_] ≥ 4.5 (prediction mean close to the upper quality limit). The prediction standard deviation $\sigma_{Qcon}$ serves only as an uncertainty indicator for comparison and interpretation, and is not treated as a hard constraint. Among solutions meeting the threshold, the one with the maximum (*d + h*) was selected as the recommended operating condition.

**Figure 3:**
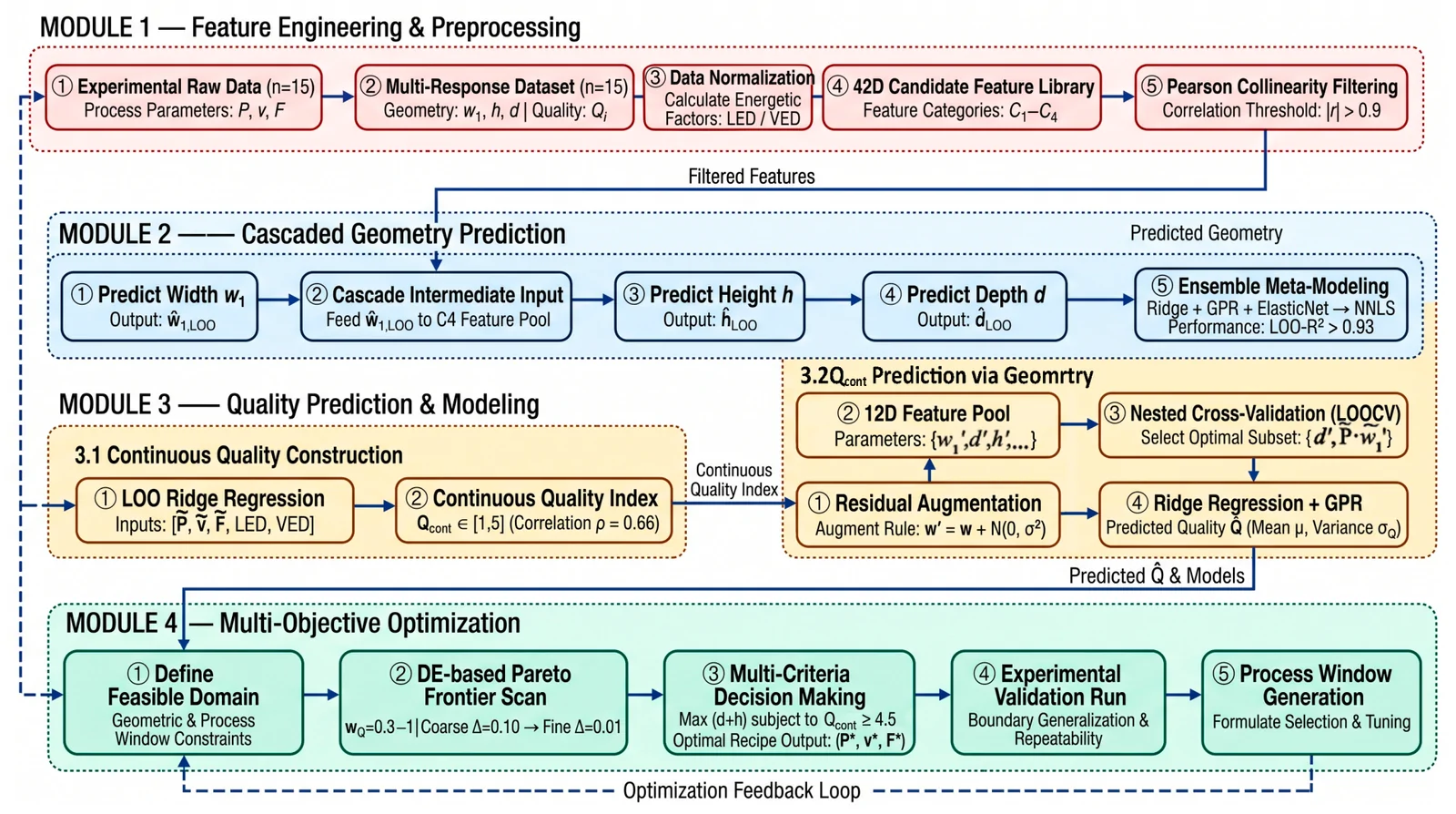
The overall methodological framework.

The overall methodological framework of this study is summarized in Figure 3.

## 3. Results and Discussion

### 3.1. Experimental Measurements and Quality-Scoring Reliability

The measured geometric responses, consensus quality grades, and corresponding sample quality weights are summarized in Table 5. The consensus grades covered the full ordinal range from 1 to 5, indicating that the experimental design included both poorly formed and high-quality tracks.

The expert evaluation demonstrated strong grading reliability. The inter-observer quadratic-weighted Cohen’s kappa reached $\kappa_{w}$ = 0.932 (95% CI: [0.815, 1.000]), with an exact agreement rate of 86.7% and 100% agreement within ±1 grade. The intra-observer reliability was similarly robust ($\kappa_{w}$ = 0.965), confirming that the established ground-truth labels (*Q*_dis_) provide a dependable foundation for quantitative modeling.

### 3.2. Verification and Physical Interpretability of the Geometry Model

#### 3.2.1. Feature Importance and Physical Interpretability (SHAP)

**Figure 4:**
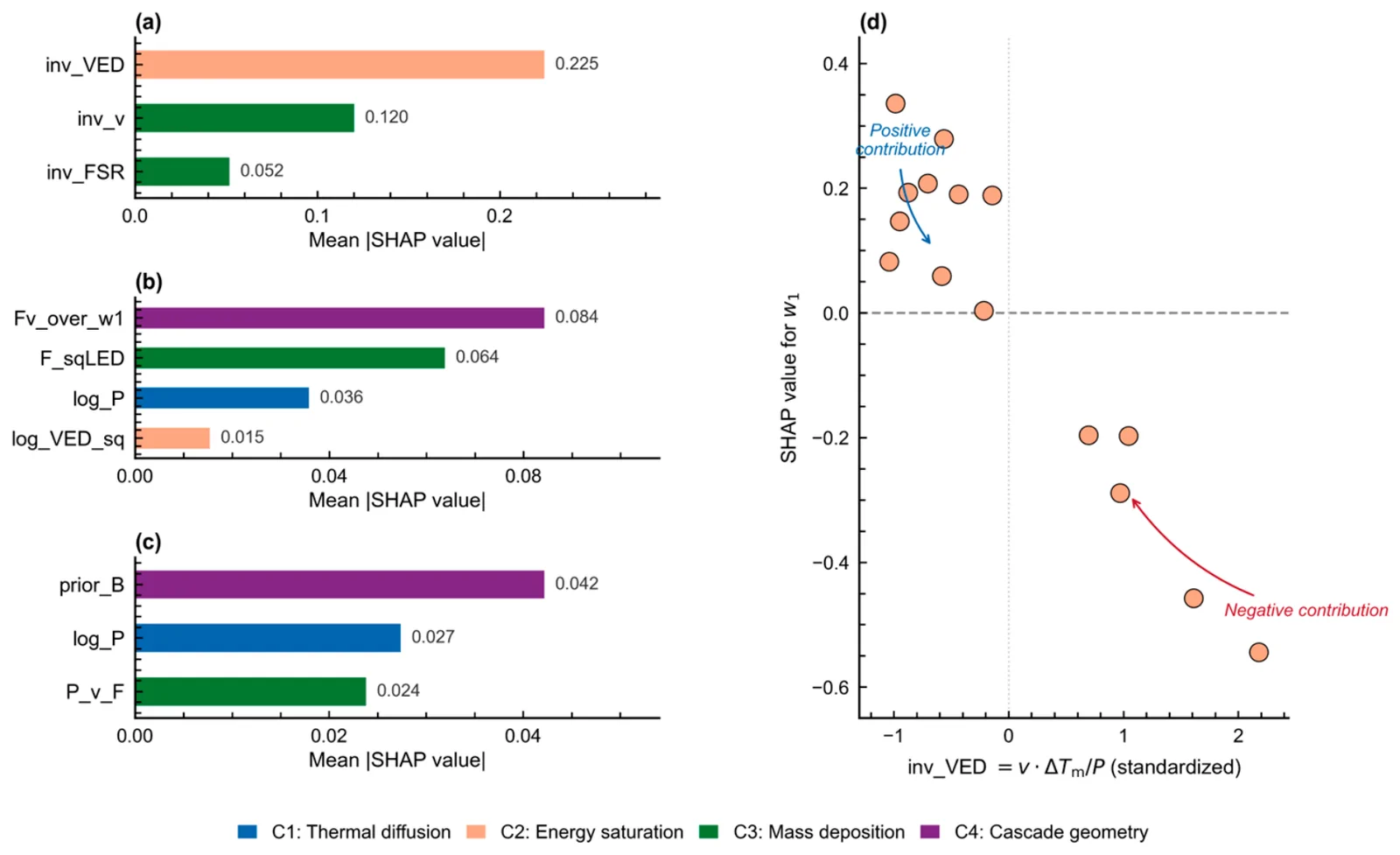
SHAP-based feature attribution analysis of key laser cladding parameters. (**a**) Clad width *w*_1._ (**b**) Clad height *h*. (**c**) Depth *d*. (**d**) inv_VED dependence on *w*_1_, each marker represents one of the 15 experimental conditions.

SHAP attribution analysis was conducted on the optimal feature subsets (Table A2) to quantify descriptor contributions and uncover the underlying physical mechanisms governing the track geometry (Figure 4). The results demonstrate that each geometric response is primarily driven by a distinct core descriptor reflecting domain-specific transport phenomena; for track width *w*_1,_ the inv_VED descriptor yields the highest mean absolute SHAP value (0.225), confirming that lateral track expansion is primarily dictated by the balance of energy density and scanning speed, which governs the extent of lateral thermal diffusion within the melt pool. In the track height model *h*, the powder deposition descriptor Fv_over_w1 serves as the dominant contributor (0.084); this reflects a rigid mass conservation constraint, wherein the track height must inversely adjust to the width and feed rate to maintain a consistent mass distribution across the deposited cross-section. For the $Ac_{1}$ depth d, the cascaded descriptor prior_B (predicted width) exhibits the leading contribution (0.042), capturing the synergistic relationship between the thermal footprint—where the melt pool width directly influences the isotherms—and the subsequent thermal conduction that establishes the transformation boundary. Collectively, these findings demonstrate that the cascaded-learning framework effectively learns the non-linear coupling between energy input saturation and material response, confirming that the identified predictive drivers are physically consistent with the governing transport phenomena of the laser cladding process.

The prominence of prior_B validates the predictive efficacy of integrating domain-informed cascade architectures into data-driven frameworks. Its persistent retention through collinearity screening and sparse subset selection confirms that the empirical data strongly support the cascade prior. The learned linear relationship between d and is expressed as

(13) $$\frac{d}{{\hat{w}}_{1}^{LOO}}\approx \kappa$$

The identified scaling coefficient, κ = 0.211, shows remarkable agreement with the theoretical aspect ratio interval (κ = 0.15–0.25) derived from the classic Rosenthal moving point heat source solution [14]. This quantitative alignment confirms that the proposed framework effectively captures the dominant thermal conduction behavior governing solid-state phase transformation boundaries.

Figure 4d further indicates that the SHAP dependence curve of inv_VED exhibits an obvious sign reversal at the zero point. High specific energy input (negative normalized values) facilitates lateral melt pool expansion, while low specific energy input (positive normalized values) yields negative contributions to clad width. This nonlinear transition originates from the energy saturation effect; energy input exceeding the critical threshold primarily deepens the melt pool rather than widening its lateral dimension.

#### 3.2.2. Model Accuracy

**Figure 5:**
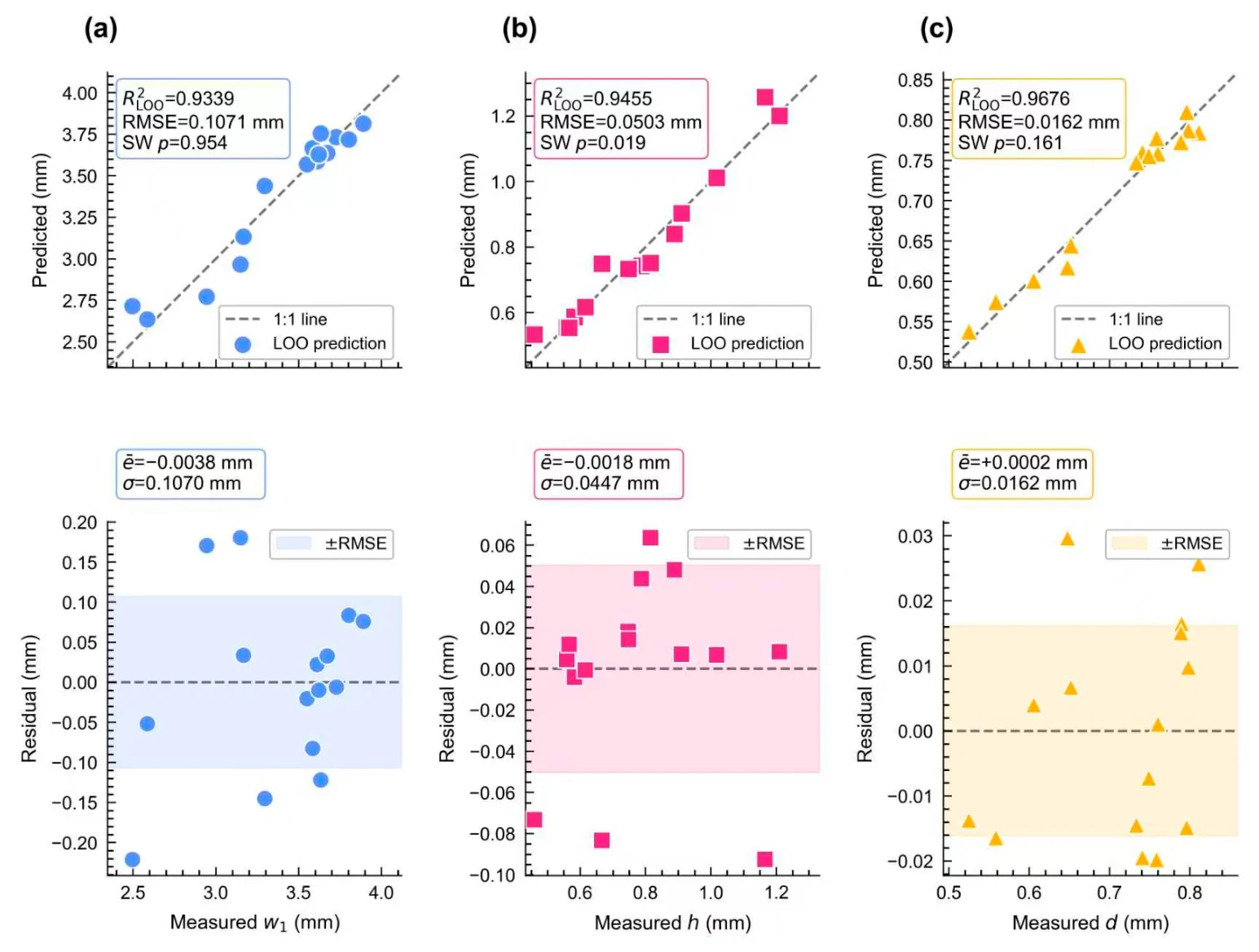
LOO-CV prediction vs. measurements (**top**) and residual analysis with Shapiro–Wilk normality test (**bottom**): (**a**) *w*_1_, (**b**) *h*, (**c**) *d*.

The LOO-CV performed on the entire experimental dataset (Figure 5) demonstrated high predictive accuracy across all targets. The out-of-fold coefficient of determination *R*^2^ reached 0.934, 0.946, and 0.968 for *w*_1_, *h*, and *d*, respectively, with corresponding RMSE values of 0.107 mm, 0.050 mm, and 0.016 mm. The mean residuals were centered near zero ($\bar{e}$ = −0.0038 mm for *w*_1_, −0.0018 mm for *h*, and 0.0002 mm for *d*), and the vast majority of data points clustered tightly within the ±RMSE bounds without discernible systematic bias.

The exploratory Shapiro–Wilk normality tests on the pooled residuals yielded *p*-values of 0.954, 0.019, and 0.167 for *w*_1_, *h*, and *d*, respectively. The residuals of *w*_1_ and *d* showed no significant departure from normality (*p* > 0.05), whereas the *h* model exhibited a minor deviation (*p* = 0.019). Considering the limited sample size (*N* = 15) and the inherent sample overlap in LOO cross-validation, these *p*-values are treated as descriptive diagnostics rather than definitive proof of normality. Together with the low error bounds and residual distributions, these results confirm the favorable statistical robustness and reliability of the overall predictive framework.

**Figure 6:**
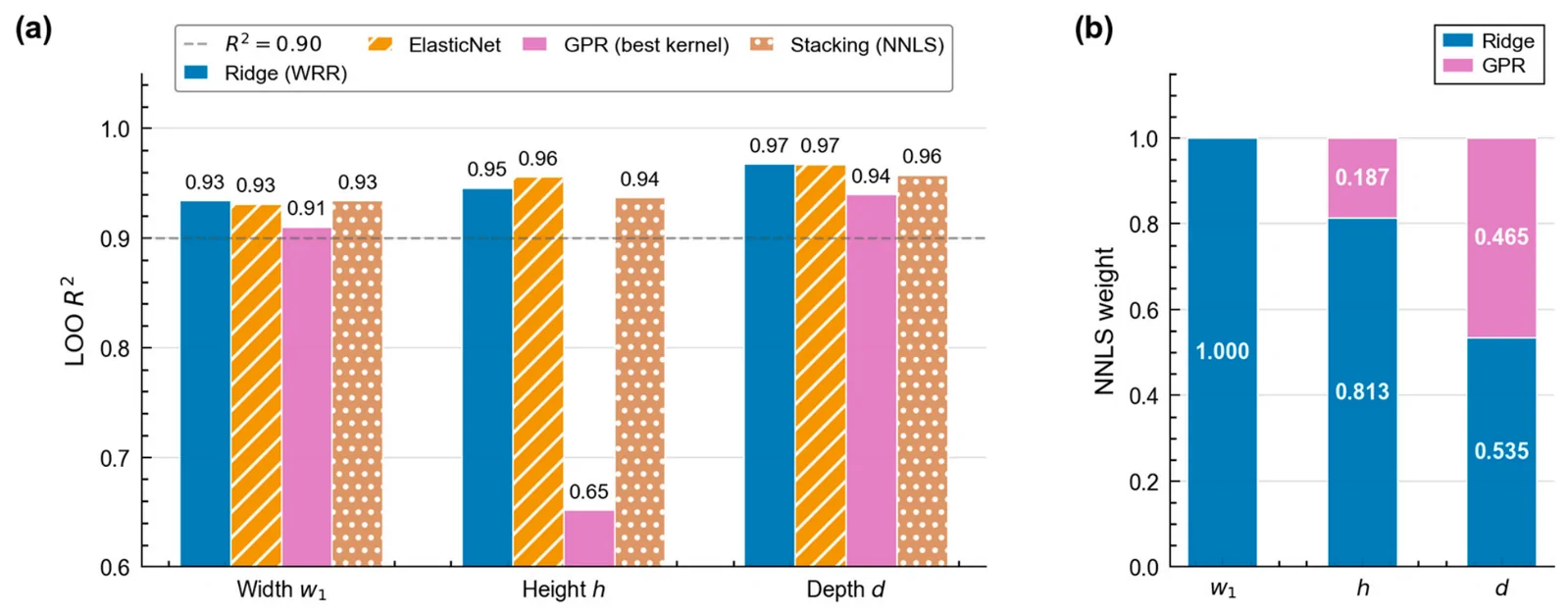
Multi-learner LOOCV comparison and NNLS stacking weights: (**a**) LOOCV comparison; (**b**) NNLS stacking weights.

Weight allocation and performance comparison of the stacking ensemble model based on NNLS (Figure 6) reveal distinct governing mechanisms for the different geometric variables. For *w*_1_, standalone Ridge regression achieves near-optimal predictive performance (LOO *R*^2^ = 0.934) and attains a unity weight of 1.000 within the ensemble. This outcome demonstrates that lateral melt pool evolution is dominated by linear physical responses. In contrast, GPR contributes weights of 0.187 and 0.465 to the ensemble predictions of *h* and *d*, respectively, which effectively compensate for nonlinear physical effects that cannot be captured by linear Ridge regression.

As shown in Figure 6a,b, Ridge regression demonstrated superior robustness across all geometric targets (R^2^ ≥ 0.93), particularly avoiding the severe performance collapse observed in GPR for track height *h* (R^2^ = 0.65). The non-negative least squares (NNLS) stacking mechanism effectively leveraged this stability by allocating dominant weights to Ridge (*w*_1_—1.000, *h*—0.813, *d*—0.535), thereby insulating the overall ensemble from the variance of non-linear base learners. Notably, although ElasticNet yielded comparable standalone accuracy (R^2^ ≥ 0.93, Figure 6a), it was assigned zero weight across all targets in the stacking ensemble. This zero-weight attribution originates from the high collinearity between the cross-validation prediction vectors of ElasticNet and Ridge, leading NNLS to prune the redundant predictive signal in favor of the strictly L_2_-regularized Ridge estimator, which delivers an optimal bias–variance tradeoff on correlated process descriptors. The resulting explicit mathematical expressions derived from Ridge regression for the geometric targets are summarized in Equation (14).

(14) $$\left\{\begin{array}{c} w_{1}=8.931-2.116\times inv\text{\_}VED-1.956\times inv\text{\_}v-1.352\times inv\text{\_}FSR\;\\ h=0.535+2.804\times Fv\text{\_}over\text{\_}w1-2.512\times F\text{\_}sqLED+3.080\times log\text{\_}p-1.472\times log\text{\_}VED\text{\_}sq\;\\ d=0.437+0.566\times prior\text{\_}B-0.126\times p\text{\_}v\text{\_}F+0.380\times log\text{\_}p\; \end{array}\right.$$

**Figure 7:**
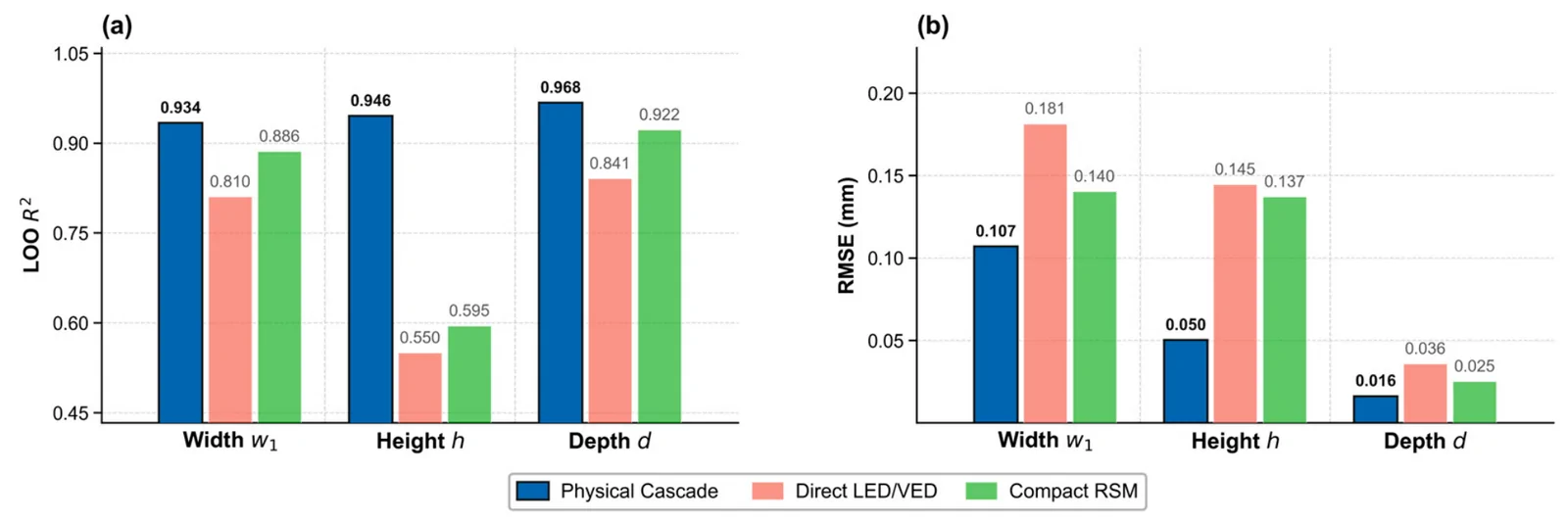
LOO cross-validation performance of three modeling strategies: (**a**) Coefficient of determination (R^2^). (**b**) Root mean square error (RMSE).Bold values indicate the best-performing method for each geometric response.

The LOOCV metrics across all three geometric responses—width (*w*_1_), height (*h*), and depth (*d*)—are quantitatively summarized in Figure 7. Across all three geometric responses, the physical cascade model consistently delivered the highest R^2^ and lowest RMSE, confirming that embedding the physics-informed cascade structure into the surrogate framework provides measurable and systematic predictive advantages over purely data-driven energy density or polynomial baselines, even under the constraint of a small 15-sample training set.

#### 3.2.3. Effects of Process Parameters on Clad Geometric Dimensions

**Figure 8:**
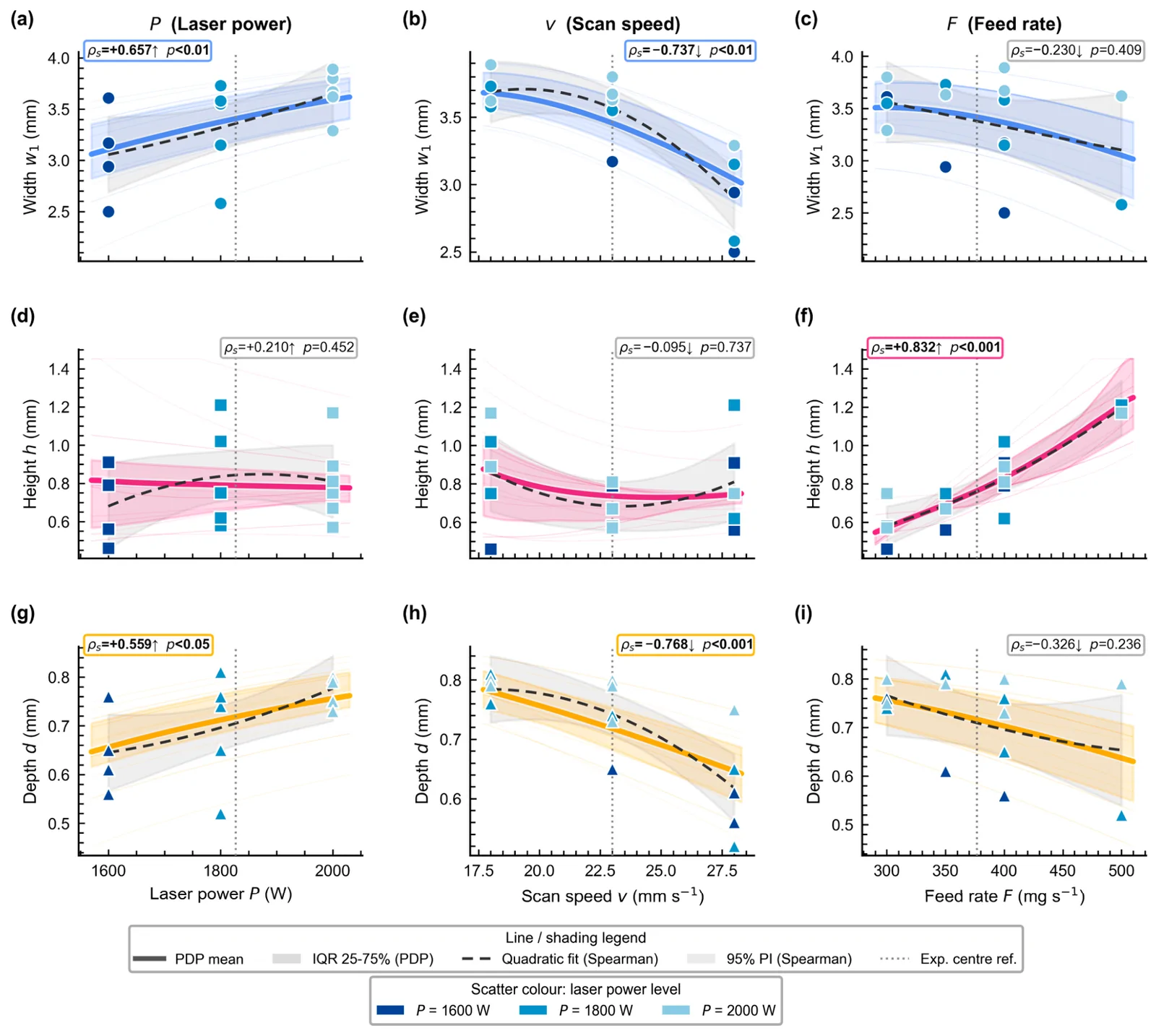
Partial dependence and individual conditional expectation curves for three responses (*w*_1_, *h*, *d*) vs. process parameters (*P*, *v*, *F*). Panels: (**a**) *w*_1_–*P*, (**b**) *w*_1_–*v*, (**c**) *w*_1_–*F*, (**d**) *h*–*P*, (**e**) *h*–*v*, (**f**) *h*–*F*, (**g**) *d*–*P*, (**h**) *d*–*v*, (**i**) *d*–*F*.

Figure 8 presents Spearman rank correlation results and partial dependence, individual conditional expectation (PDP/ICE) marginal response curves that quantify how processing parameters (*P*, *v*, *F*) affect melt pool geometric metrics (*w*_1_, *h*, *d*). Correlation analysis reveals that clad width *w*_1_ and *Ac*_1_ boundary depth *d* are predominantly governed by energy input ($\rho_{s}$ ≥ 0.559, *p* < 0.05), with monotonic response trends against their dominant predictors. This observation confirms that linear energy density (LED = *P*/*v*) serves as the dominant physical factor governing thermal penetration and lateral melt pool expansion. In contrast, clad height *h* follows clear mass-conservation behavior and exhibits an extremely strong positive correlation with powder feed rate F ($\rho_{s}$ = +0.832, *p* < 0.001), while thermal input parameters (*P*, *v*) exert only minor influences on *h*.

**Figure 9:**
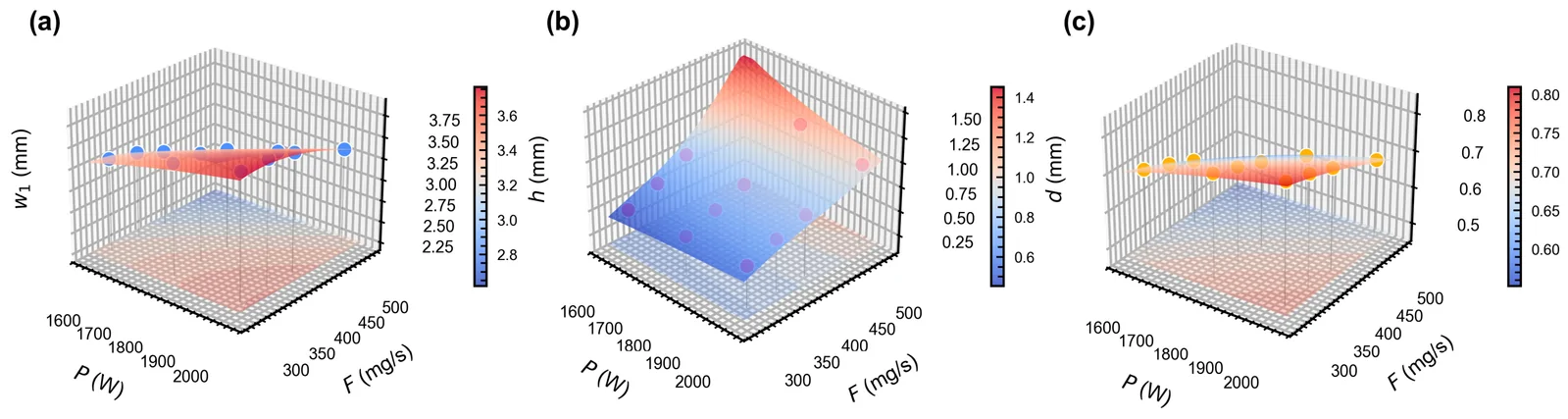
Three-dimensional response surfaces in the *p*–*F* plane at *v* = 23 mm s^−1^ (experimental center). (**a**) *w*_1_, (**b**) *h*, (**c**) *d*.

To provide a more intuitive visualization of the interactions, a 3D response surface analysis was performed at a constant scan speed of *v* = 23 mm/s (Figure 9). The analysis indicates that while *h* increases monotonically with *F*—consistent with a mass-conservation-governed deposition mechanism—the geometric responses *w*_1_ and *d* exhibit more complex dependencies. Although their sensitivities to *F* are generally lower than that of *h*, it is evident that these sensitivities are coupled with the laser power *P*. Specifically, at lower power levels (1600 W), *w*_1_ and *d* show a visible decline as *F* increases, likely due to the enhanced thermal shielding and energy absorption achieved by the powder stream. As *P* increases, the dominance of energy input in driving thermal penetration and lateral expansion tends to mask this effect, leading to the observed ‘flatter’ surface at higher power ranges. This confirms a dual control mechanism, whereby *h* is primarily constrained by mass flux, whereas *w*_1_ and *d* are governed by the interaction between energy input and the secondary effects of powder-related thermal dissipation.

Taken together, the Spearman correlation, PDP/ICE, SHAP, and response surface results indicate a partially decoupled control structure rather than complete independence among the processing parameters. Laser power *P* and scanning speed *v* primarily define a thermal-input channel governing *w*_1_ and *d* (*p* < 0.01), whereas the powder input per unit track length, (*F*/*v*), forms a mass-addition channel that predominantly controls (h). This interpretation is consistent with those of Kermani et al. [20] and Qi et al. [48], who found that track width was governed mainly by energy input, whereas track height was more sensitive to powder addition. Nevertheless, the two control channels remain coupled. At (*P* = 1600) W, increasing (*F*) visibly decreases (*w*_1_) and (*d*), probably because powder attenuation and the reduced energy available per unit of deposited mass become more influential. Similar interaction-dependent effects on heat-affected zone geometry were reported by Yu et al. [49]. The identified control relationships should therefore be interpreted within the present material, equipment, and parameter ranges, rather than as universally transferable sensitivities.

### 3.3. Performance and Fidelity of the Continuous Quality Prediction Model

To evaluate the numerical behavior and fidelity of the continuous auxiliary representation, predictive models (regularized Ridge and Gaussian Process Regression, GPR) were evaluated against both continuous (*Q*_cont_) and discrete consensus (*Q*_dis_) targets using identical geometric/dimensionless features ($d^{\prime }$, $\tilde{p}\cdot \tilde{{w_{1}}^{\prime }}$). The predictive performance is summarized in Table 6. To enable a direct comparison across representations with the same effective span ([1, 5], data range = 4), normalized LOO MAE metrics were computed.

Normalized results show that for the linear Ridge model, the normalized MAE decreases from 15.0% on the discrete target to 10.25% on the continuous scale, corresponding to a relative reduction of 31.7%. For the nonlinear GPR model, the normalized MAE drops from 18.25% to 9.25% (49.3% relative reduction). Under the continuous formulation, the LOOCV R^2^ values reach 0.82 for Ridge and 0.84 for GPR, demonstrating high variance explanation, which is consistent with the substantial inter-rater reliability observed in the discrete domain (QWK of 0.76 for Ridge and 0.72 for GPR).

It should be acknowledged that because the continuous target *Q*_cont_ is inherently a smoother manifold derived from physical geometric trends, part of this MAE reduction is structurally built in due to the elimination of discrete quantization boundaries and ordinal step penalties. Rather than claiming absolute algorithmic superiority over discrete classification, this transition confirms that *Q*_cont_ provides a well-conditioned, numerically stable landscape suitable for downstream multi-objective optimization, while GPR effectively captures residual nonlinearities.

The expression of the fitted Ridge regression model is given by

(15) $$Q_{cont}=8.759\cdot d^{\prime }+1.437\cdot \tilde{P}\cdot \tilde{w_{1}^{\prime }}-4.127$$

The large positive regression coefficient of *d*′ indicates that cladding quality improves markedly with the increasing penetration depth of the Ac_1_ isotherm. This parameter reflects the effective HAZ of the substrate and the extent of thermal conduction from the melt pool into the base material. A deeper Ac_1_ isotherm generally corresponds to more sufficient energy input and the stronger thermal effect of the melt pool, which facilitates powder melting, molten droplet spreading and material deposition. On the other hand, the interaction term $\tilde{p}$⋅$\tilde{{w_{1}}^{\prime }}$ also shows a positive contribution, indicating that the effect of laser power on cladding quality is not independent but synergistically modulated by the transverse dimension of the clad track. Under wider clad track conditions, increasing laser power further enlarges the effective melt pool volume and powder capture efficiency, thus enhancing the deposition quality of a single track. These results reveal that cladding quality is governed by the coupling of heat input intensity and melt pool geometry, and the Ac_1_ penetration depth serves as a critical thermal characteristic parameter reflecting the heat and mass transfer behavior during laser cladding.

### 3.4. Multi-Objective Trade-Off and Experimental Validation

**Figure 10:**
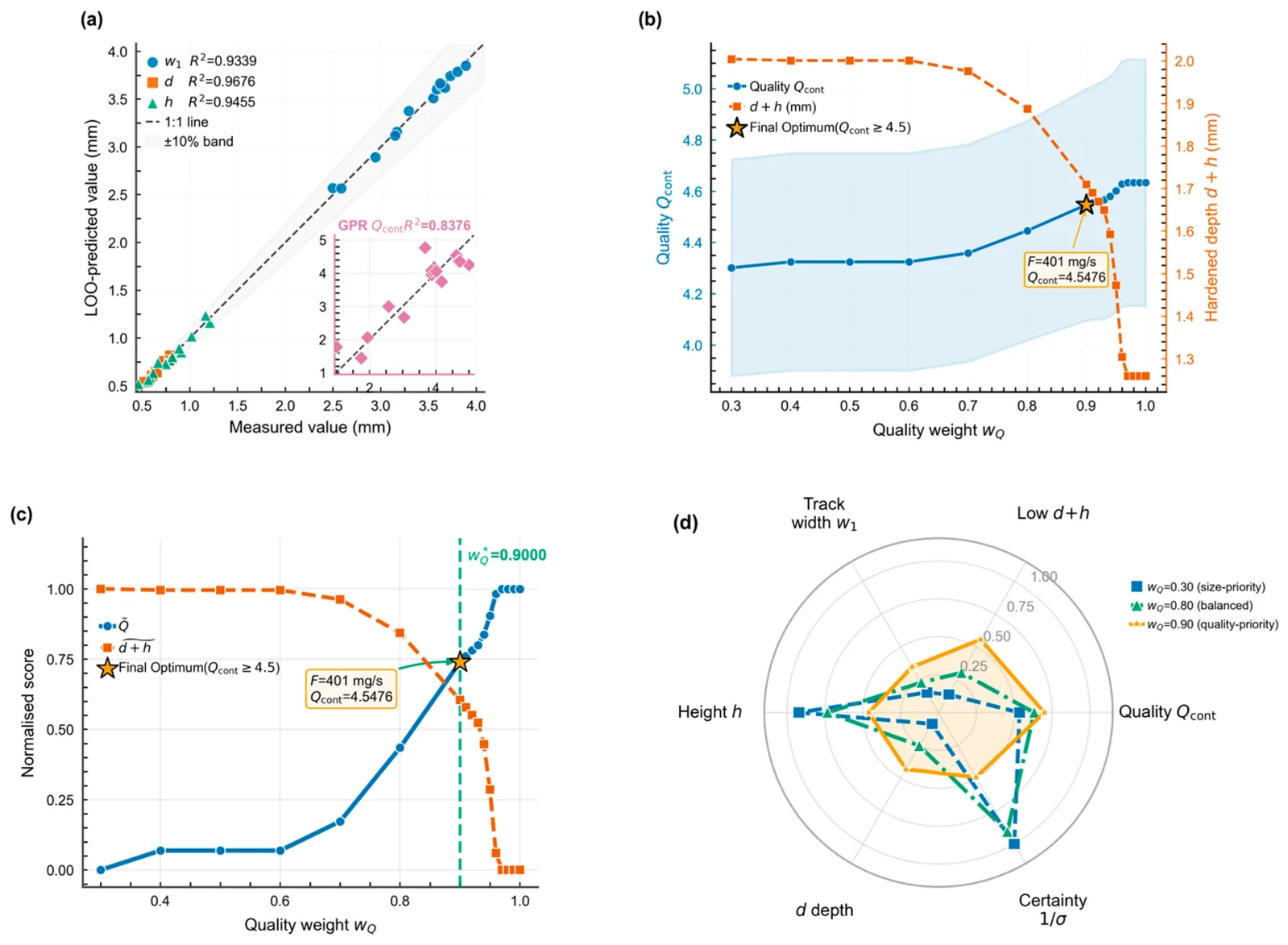
Multi-objective Pareto optimization: (**a**) Model fidelity; (**b**) trade-off between quality and hardened depth; (**c**) normalized bi-objective curves; (**d**) multi-performance radar chart.

Leave-one-out (LOO) cross-validation confirmed the predictive fidelity of the surrogate framework (Figure 10a). Geometric outputs (*w*_1_, *d*, *h*) tracked the 1:1 line within ±10% ($R^{2}$= 0.9339, 0.9676, 0.9455), while the continuous quality metric *Q*_cont_ achieved $R^{2}$ = 0.8376, which is lower than the geometric outputs, consistent with the coarser resolution of the original discrete quality scoring. This fidelity justified extending the surrogate beyond the discrete experimental dataset to enable DE-based continuous-space multi-objective optimization.

Scanning the quality weight $w_{Q}$ revealed a nonlinear trade-off between *Q*_cont_ and (*d* + *h*) (Figure 10b,c), namely, for $w_{Q}$ ≤ 0.6, the solution remained essentially invariant, while beyond $w_{Q}$ ≈ 0.7, further increases in $w_{Q}$ progressively reduced powder flux *F*, raising *Q*_cont_ at the expense of (*d + h*). Enforcing *Q*_cont_ ≥ 4.5 as a quality constraint to ensure acceptable clad quality, $w_{Q}$ = 0.90 was selected as the optimal setting, yielding *Q*_cont_ = 4.5476 (σ = 0.4524) with *F* ≈ 401 mg s^−1^ (*P* = 2000 W, *v* = 18 mm s^−1^) and (*d* + *h*) = 1.710 mm. A radar comparison at $w_{Q}$ = 0.30, 0.80 and 0.90 (Figure 10d) showed that the quality priority setting retained a large overall radar area, indicating a balanced extremum across correlated metrics rather than single-axis dominance—supporting $w_{Q}$ = 0.90 as a globally, rather than locally, optimal solution.

For experimental implementation, the predicted powder feed rate was rounded by 1 mg s^−1^ to the nearest attainable machine setting, *F* = 400 mg s^−1^. This minor adjustment was treated as negligible relative to the experimental control resolution. Crucially, this optimal condition (*P* = 2000 W, *v* = 18 mm s−1, *F* = 400 mg s^−1^) coincided with discrete training sample No. 13 (*Q* = 5) from the initial training dataset. The identification of this experimentally realized joint optimum supports the physical consistency of the surrogate-guided multi-objective optimization. However, because the condition was included in the training set, this agreement alone does not constitute independent evidence against over-fitting.

The model was therefore assessed using two complementary experiments. No. 16 was a previously unseen boundary condition (*P* = 1600 W, *v* = 23 mm s^−1^ and *F* = 300 mg s^−1^) used to assess out-of-sample generalization. No. 17 was an independent-batch replicate of training condition No. 13, and was used to assess the repeatability of the experimentally implemented optimum.

**Figure 11:**
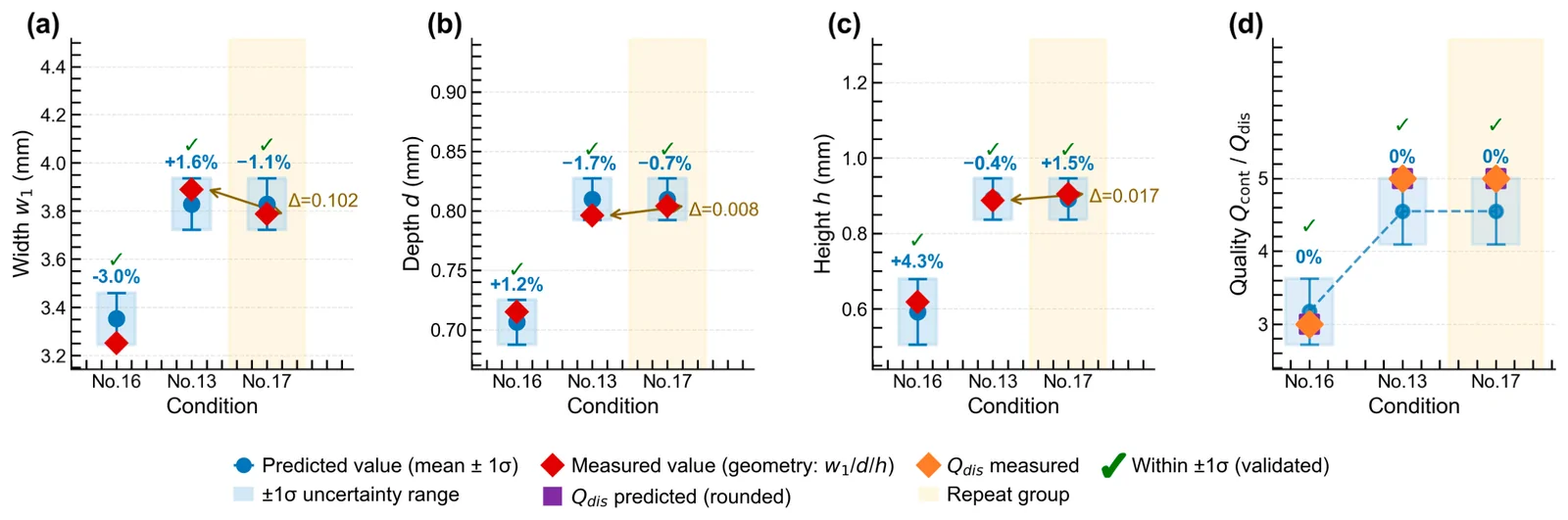
Quantitative validation of the surrogate framework across generalization and repeatability regimes. Comparison between model predictions and experimental measurements for (**a**) *w*_1_, (**b**) *d*, (**c**) *h*, (**d**) *Q*_cont_ and *Q*_dis._

**Figure A2:**
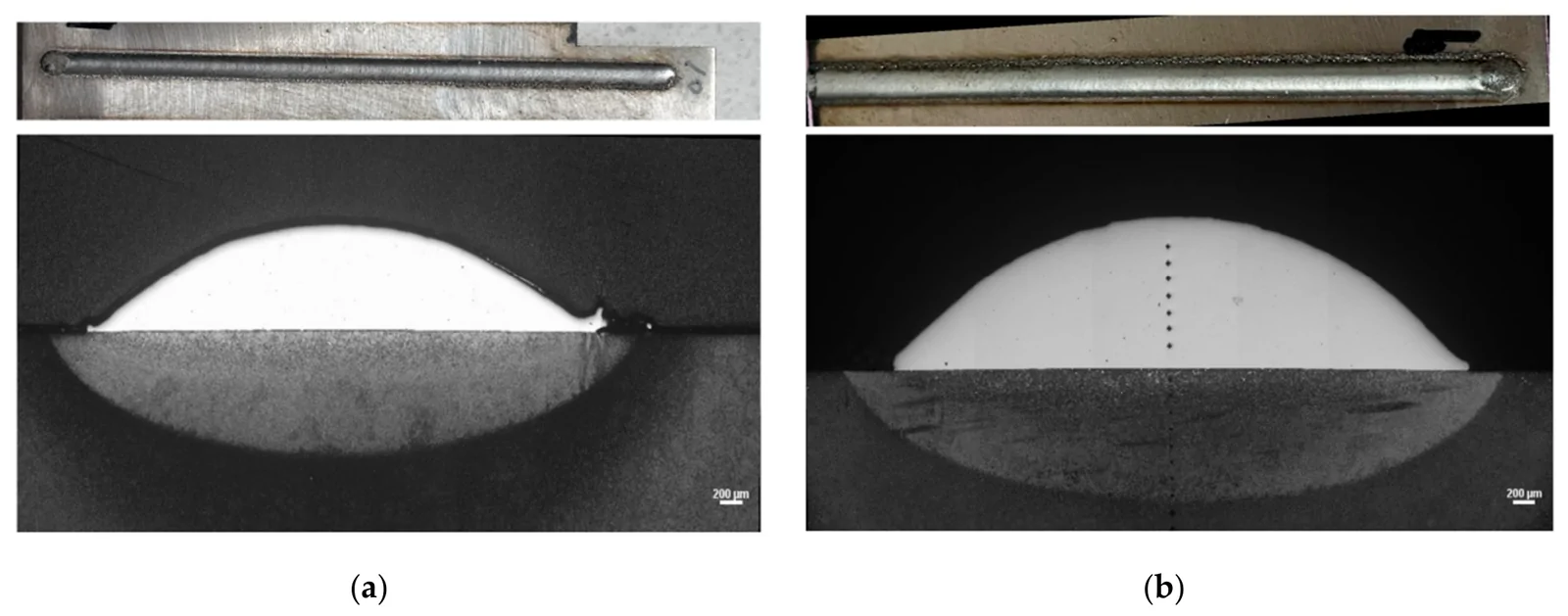
Characterization results of the verification experiment. (**a**) Sample 16: *P* = 1600 W, *v* = 23 mm/s, *F* = 300 mg/s. (**b**) Specimen 17: *P* = 2000 W, *v* = 18 mm/s, *F* = 400 mg/s.

A direct comparison between the experimentally measured geometries and the cascade model predictions is illustrated in Figure 11 (micro-structural validation is detailed in Appendix A, Figure A2). As shown in Figure 11a–c, all measurements fell within the model’s ±1σ uncertainty bands.

Under the genuine generalization test (No. 16), the relative prediction errors for *w*_1_, *d*, and *h* were tightly bounded at −3.0%, +1.2%, and +4.3%, respectively. This result supports the predictive validity of the physics-informed cascade model at this unseen boundary condition.

Under the repeatability test (No. 17 vs. No. 13), batch-to-batch variation was minimal (Δw_1_ = 0.102 mm, Δd = 0.008 mm, Δh = 0.017 mm), and the discrete quality index *Q* remained consistent across replicates (Figure 11d). These differences were comparable to or smaller than the corresponding model-reported predictive uncertainties, supporting the batch-to-batch repeatability of the selected operating condition.

Taken together, the No. 16 experiment provides an external predictive check at one previously unseen boundary condition, whereas the No. 17 experiment demonstrates the repeatability of the experimentally implemented constrained optimum across two fabrication batches. The results therefore identify conditions No. 13/17 as a reproducible candidate operating point that maximizes the predicted (d+h) while satisfying $\left(Q_{cont}\geq 4.5\right)$ within the investigated design space. Additional experiments spanning multiple batches, material lots and operating environments would nevertheless be required before production-scale robustness or direct scale-up could be established.

The uncertainty estimates should nevertheless be interpreted as model-derived ranking indicators rather than calibrated engineering limits. In each LOO fold, the GPR and residual dispersion estimates were derived from only 14 training observations, and were conditional on the selected model structures. The σ terms were therefore used only as a soft penalty to discourage poorly supported candidates, whereas the process parameter bounds and ($Q_{cont}\geq 4.5$) defined feasibility. The inclusion of the No. 16 and No. 17 measurements within the model-reported (±1σ) bands provides a consistency check at these conditions, but does not establish the nominal coverage probability of the intervals. Larger multi-batch datasets will be required to calibrate predictive coverage and establish engineering process capability limits.

### 3.5. Process Window Delineation

**Figure 12:**
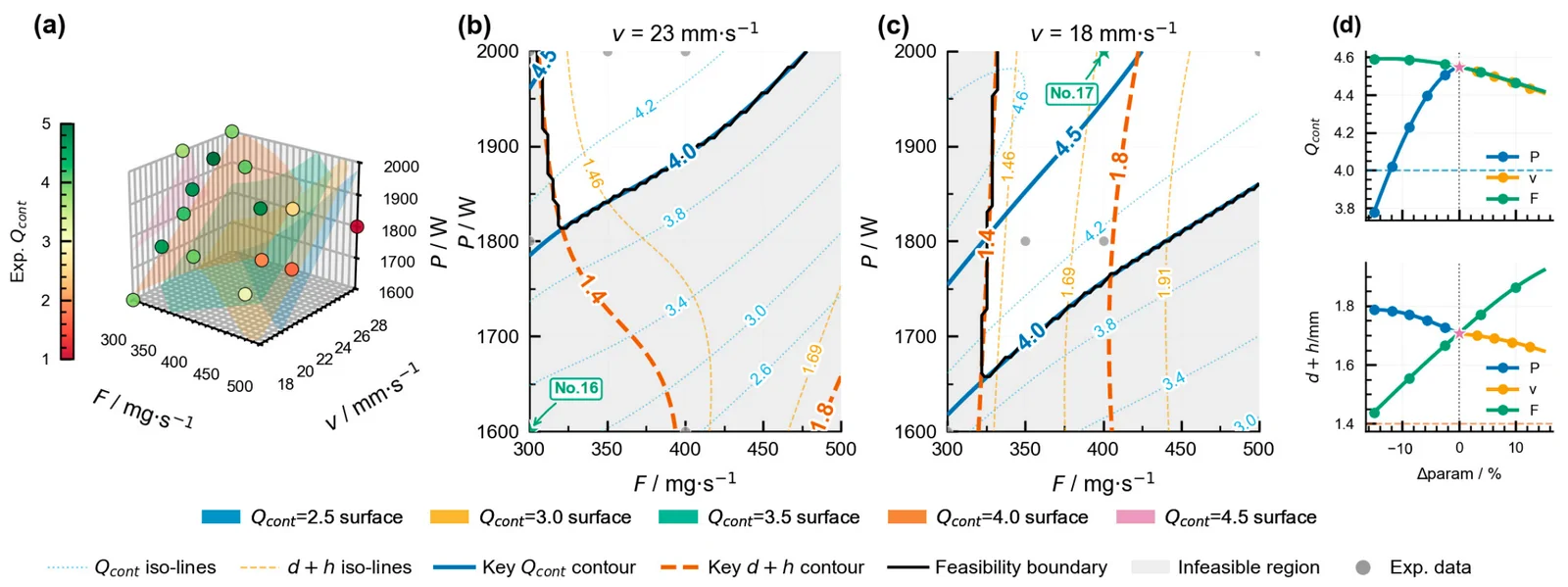
Multi-response constraint map: *Q*_cont_ and geometry (*w*_1_, *d*, *h*). (**a**) 3D isosurfaces of *Q*_cont_; (**b**) *v* = 23 mm s^−1^ slice with feasible region and thresholds; (**c**) *v* = 18 mm s^−1^ slice with feasible region and thresholds; (**d**) *P*, *v*, *F* sensitivities for the two responses at the reference point.

To establish a process window for the single-track laser-cladding repair of carburized-quenched steel, a multi-criterion process map was constructed in the three-dimensional *P–v–F* space (Figure 12) using the predicted geometric responses, $A_{c1}$ boundary depth *d*, and continuous quality response. The experimental ordinal quality grades were represented by $Q_{cont}$, and the predicted quality distribution was visualized using isosurfaces. Figure 12a presents the isosurfaces of *Q*_cont_ = 2.5–4.5 and the corresponding experimental data points (color-coded by *Q*_cont_), which clearly demonstrate the attainable quality range and its gradient driven by the combined effects of *P*, *v* and *F*.

For engineering feasibility assessment, joint constraints were applied to *Q*_cont_ and the combined depth (*d + h*), and the resulting boundaries were projected onto slices at fixed scanning speeds (Figure 12b,c). At *v* = 23 mm/s (Figure 12b), *Q*_cont_ ≥ 4.0 and (*d + h*) ≥ 1.4 mm constitute the basic engineering thresholds. The feasibility boundary (solid black line) clearly separates parameter combinations that satisfy the constraints from the infeasible region (shaded in gray). Contour lines of *Q*_cont_ and (*d + h*) are superimposed to characterize the margin within the window, and a representative boundary experimental point (No. 16) is marked, which lies in the under-constrained region.

At *v* = 18 mm/s (Figure 12c), in addition to the (*d + h*) ≥ 1.4 mm constraint, a threshold line of *Q*_cont_ ≥ 4.5 is further provided to characterize the shrinkage and shift of the process window under tighter constraints. Accordingly, point No. 17 satisfies both the quality and (*d + h)* requirements on this velocity slice.

Overall, by superimposing the threshold constraints of *Q*_cont_ and (*d + h*), the process map explicitly delineates the feasible operation domain and provides directional guidance for parameter optimization toward higher quality and larger effective hardening depth. Nevertheless, the feasible region boundary is static, while practical cladding operations are inevitably affected by parameter setting deviations and process disturbances. To evaluate process robustness, a local sensitivity analysis was performed on the *v* = 18 mm/s slice (inset in Figure 11c), where the responses of *Q*_cont_ and (*d + h)* to *p*, *v* and *F* were investigated within a perturbation range of δ_param = ±10%.

The sensitivity results indicate that near this operating condition, *Q*_cont_ exhibits the strongest response to laser power *p*, while being less sensitive to scanning speed *v* and powder feed rate *F*. In contrast, (*d + h*) is more sensitive to *F*, suggesting that the powder feed rate dominates the dimensional evolution of the deposited layer.

The combined process maps translate these relationships into a directional parameter-adjustment strategy. The required limits for $Q_{cont}$ and the combined depth, (*d* + *h*), are first specified, after which an operating condition is selected inside, rather than directly on, the feasible boundary to retain a tolerance margin. Within the investigated process window, *P* is the primary corrective variable when $Q_{cont}$, *w*_1_, or *d* is insufficient; alternatively, *v* may be reduced when permitted by productivity and heat-input constraints. Once an adequate quality margin has been established, *F* can be used primarily to adjust *h* and *d* + *h*. At low power, however, increasing *F* may require a corresponding increase in available energy to prevent the deterioration of *w*_1_, *d*, or morphological quality. Conversely, excessive clad height should preferentially be corrected by reducing *F* or increasing *v*. Compared with conventional process maps based mainly on bead geometry, dilution, deposition rate, or powder efficiency [8,20], the present maps additionally incorporate morphological quality and the $A_{c1}$ boundary depth. They therefore provide an application-oriented basis for parameter screening and directional adjustment in the laser-cladding repair of carburized gear steel, while the quantitative process boundaries remain specific to the present material and equipment.

## 4. Conclusions

(1) A physics-informed cascaded-learning framework was developed to predict track width *w*_1_, clad height *h*, and the $A_{c1}$ boundary depth *d*. Leakage-controlled out-of-fold width predictions were transferred to the downstream height and depth models, representing the hierarchy $w_{1}\to h\to d$. The geometric models achieved LOOCV (R^2^) values of 0.934–0.968. At the previously unseen boundary condition No. 16, the maximum relative prediction error was 4.3%, supporting the predictive capability of the framework at this external test point.
(2) Interpretability analyses identified two dominant but coupled control channels. The energy-input channel, governed primarily by *p*/*v*, controlled $w_{1}$, *d* and morphological quality, whereas the mass-addition channel, represented by *F*/*v*, predominantly controlled *h*. Within the investigated process window, *P* was therefore the primary corrective variable for insufficient quality, width, or boundary depth, while *F* principally adjusted *h* and (*d* + *h*). Because changing *v* simultaneously alters both energy and powder input per unit length, it is less response-selective. Moreover, increasing *F* at low power may reduce the energy available per unit deposited mass; thus, energy–mass coupling must be retained during parameter adjustment.
(3) An auxiliary continuous quality response provided a smooth landscape for constrained optimization, while the observed ordinal grades were retained as reference labels. Maximizing the predicted (*d* + *h*) under $Q_{cont}\geq 4.5$ recovered the experimentally realized condition *P* = 2000 W, *v* = 18.0 mm s^−1^, and *F* = 400 mg s^−1^. This result represents the recovery of a constrained operating point within the investigated design space rather than the discovery of a previously untested global optimum. The independent-batch replicate No. 17 showed only minor geometric variation and an unchanged ordinal quality grade, supporting the repeatability of the implemented operating condition.
(4) More broadly, the study demonstrates a transparent route for integrating physical descriptors, leakage-controlled cascading, interpretable learning, and constrained process maps in small-sample laser cladding development. The quantitative boundaries remain specific to the present material, equipment, and single-track dataset. Future work should extend the framework to larger multi-batch datasets, multi-track deposition, calibrated uncertainty, and in situ monitoring. Mechanical and fatigue validation will also be required to link the predicted responses directly to component-level service performance.

## Figures and Tables

**Table 1:** Chemical composition (wt.%) of 18CrNiMo7-6 substrate.

Material	C	Cr	Ni	Mo	Mn	Si	*p*	S	Fe
18CrNiMo7-6	0.7~0.8 (surface)	1.8	1.5	0.3	0.76	0.29	0.0072	0.002	Balance

**Table 2:** Chemical composition (wt.%) of the laser-cladding powder.

Material	C	B	Cr	Ni	Mo	Mn	Si	V	Fe
NHT.22. A01	0.7	0.9	15.5	1.7	0.4	0.3	0.9	1.2	Balance

**Table 3:** Processing parameters for the 15 experimental conditions.

No.	Laser Power *P* (W)	Scanning Speed *v* (mm/s)	Powder Feed Rate *F* (mg/s)
1	1600	18	300
2	1600	28	350
3	1600	23	400
4	1600	28	400
5	1800	23	300
6	1800	18	350
7	1800	18	400
8	1800	28	400
9	1800	28	500
10	2000	28	300
11	2000	23	300
12	2000	23	350
13	2000	18	400
14	2000	23	400
15	2000	18	500

**Table 4:** Operational rubric for the five-level morphological quality assessment of single clad tracks.

Grade (*Q*)	Macroscopic Track Morphology	Powder Adhesion at the Fusion Boundary	Cross-Sectional Profile and Toe Wetting
1 (Poor)	Discontinuous track or severe balling/humping that dominates the observed track segment.	Extensive or contiguous clusters of partially melted powder along the fusion boundary.	Severe asymmetry, pronounced undercut, or sharp and poorly wetted clad toes.
2 (Fair)	Continuous track with obvious width fluctuation, recurrent humping, or marked edge roughness.	Multiple clearly visible clusters of partially melted powder along the boundary.	Clear asymmetry, inadequate toe wetting, or localized undercut.
3 (Acceptable)	Continuous track with moderate ripples or localized surface irregularities, but without severe balling or discontinuity.	Scattered and isolated partially melted powder particles.	Continuous metallurgical bonding, moderate symmetry, and no severe undercut.
4 (Good)	Stable track width and a generally smooth surface with only slight ripples or edge irregularities.	Occasional isolated powder particles.	Approximately symmetric profile with smooth toe transitions and no evident undercut.
5 (Excellent)	Highly uniform and continuous track with no visible balling, humping, or macroscopic surface defects.	No adhered powder particles visible at the specified inspection magnification.	Symmetric and smooth profile with well-wetted toe transitions and no visible undercut.

**Table 5:** Measured geometry and consensus quality grades for the 15 conditions.

No.	Width *w*_1_ (mm)	Height *h* (mm)	Depth *d* (mm)	Quality Q_dis_
1	3.61	0.46	0.76	3
2	2.94	0.56	0.61	3
3	3.17	0.79	0.65	3
4	2.50	0.91	0.56	1
5	3.55	0.58	0.74	3
6	3.73	0.75	0.81	4
7	3.58	1.02	0.76	5
8	3.15	0.62	0.65	2
9	2.58	1.21	0.52	1
10	3.29	0.75	0.75	4
11	3.80	0.57	0.80	5
12	3.63	0.67	0.79	4
13	3.89	0.89	0.80	5
14	3.67	0.81	0.73	5
15	3.62	1.17	0.79	2

**Table 6:** Continuous and discrete evaluation of the quality prediction model.

Object	Metrics	Ridge	GPR
Continuous quality *Q*_cont_	LOO R^2^	0.82	0.84
	LOO MAE	0.41	0.37
	Normalized LOO MAE * (%)	10.25	9.25
Discrete quality *Q*	QWK	0.76	0.72
	LOO MAE	0.6	0.73
	Normalized LOO MAE * (%)	15	18.25

* Normalized MAE (%) = (MAE/Data range) × 100%. The data range in this study is 4.

## Data Availability

The original contributions presented in this study are included in the article. Further inquiries can be directed to the corresponding author.

## Appendix B

**Table A1:** Feature library (C1–C4) constructed from raw process parameters *P*, *v*, *F*.

Descriptor ID	Expression	Physical Rationale	Final Model
C1—Rosenthal thermal diffusion and scaling proxies			
C1-01	$\ln\left(LED\right)$	Log linear energy density	-
C1-02	$\ln\left(VED\right)$	Log volumetric energy density	-
C1-03	$\sqrt{LED}=\sqrt{\tilde{P}/\tilde{v}}$	Square root LED (Rosenthal melt-pool width scaling)	-
C1-04	${LED}^{0.46}$	Fractional exponent LED	-
C1-05	${\tilde{P}}^{2/3}/{\tilde{v}}^{1/3}$	Two-thirds power scaling law	-
C1-06	$\tilde{P}/{\tilde{v}}^{2}$	Power per speed squared (high-speed thermal penalty)	-
C1-07	$\ln\left(\tilde{P}\right)$	Log normalized power	Height *h*: log_*p*; Depth *d*: log_*p*
C1-08	$\ln\left(\tilde{v}\right)$	Log normalized speed	-
C1-09	$\ln\left(\tilde{F}\right)$	Log normalised feed rate	-
C1-10	$\ln\left(\tilde{P}\tilde{v}\right)$	Log power speed product	-
C2—Energy saturation and nonlinearity			
C2-01	1/LED	Inverse LED	-
C2-02	1/VED	Inverse VED (solidification rate proxy)	Width w_1_: inv_VED
C2-03	${\left[\ln\left(LED\right)\right]}^{2}$	Log-LED squared	-
C2-04	${\left[\ln\left(VED\right)\right]}^{2}$	Log-VED squared (asymmetric energy nonlinearity)	Height *h*: log_VED_sq
C2-05	$\ln\left(\tilde{P}\tilde{v}\tilde{F}\right)$	Log three-parameter coupling	-
C3—Mass deposition and process parameter coupling			
C3-01	$\tilde{F}/\sqrt{\tilde{P}}$	Feed rate per square root power	-
C3-02	$\tilde{F}\cdot LED$	Feed-weighted linear energy density	-
C3-03	$\tilde{F}\sqrt{LED}$	$\mathrm{Powder}\;\mathrm{flux}\;\times \;\sqrt{LED}$: nonlinear mass-energy coupling	Height *h*: F_sqLED
C3-04	$\tilde{v}\tilde{F}$	Speed feed product	-
C3-05	$\tilde{P}\tilde{F}$	Power feed product	-
C3-06	$\tilde{P}/\tilde{F}$	Power-to-feed ratio	-
C3-07	$\ln\left(\tilde{P}/\tilde{F}\right)$	Log power-to-feed ratio	-
C3-08	$\tilde{F}/\tilde{v}$	Mass per unit track length	-
C3-09	$\tilde{v}/\tilde{F}$	Inverse feed speed ratio	Width w_1_: inv_FSR
C3-10	$\ln\left(\tilde{F}/\tilde{v}\right)$	Log mass per unit length	-
C3-11	$\sqrt{\tilde{F}/\tilde{v}}$	Square root feed speed ratio	-
C3-12	${\tilde{F}}^{1/3}/{\tilde{v}}^{2/3}$	Cube root scaled flux	-
C3-13	$\tilde{F}/{\tilde{v}}^{2}$	Feed per speed squared	-
C3-14	$\tilde{F}\cdot VED$	Feed-weighted VED	-
C3-15	$1/\tilde{v}$	Inverse scan speed	Width w_1_: inv_v
C3-16	$1/{\tilde{v}}^{2}$	Inverse speed squared	-
C3-17	$\tilde{P}\tilde{v}\tilde{F}$	Three-parameter mass energy coupling	Depth *d*: *p*_v_F
C3-18	$\tilde{P}\sqrt{\tilde{F}/\tilde{v}}$	$\mathrm{Power}\;\times \;\sqrt{flux}$	-
C3-19	${\tilde{P}}^{1/3}{\tilde{F}}^{2/3}$	Fractional exponent power feed coupling	-
C4—Cascade geometry and depth prior features			
C4-01	$\tilde{F}/\left(\tilde{v}{\hat{w_{1}}}^{LOO}\right)$	Width-normalized powder flux: mass-conservation cross-section constraint	Height *h*: Fv_over_w1
C4-02	${\hat{w_{1}}}^{LOO}$	LOO-predicted width: direct geometric prior	-
C4-03	$\ln\left(\tilde{F}/\left(\tilde{v}{\hat{w_{1}}}^{LOO}\right)\right)$	Log width-normalized flux	-
C4-04	$\sqrt{LED}\cdot s_{c}$, $s_{c}$ = 0.7055	Rosenthal thermal-scaled depth prior (OLS fit of sqrt(LED) onto *d*)	-
C4-05	$\kappa {\hat{w_{1}}}^{LOO},\kappa =0.211$	Geometric depth prior from LOO width (*d*/*w*_1_ ≈ *κ* from Rosenthal theory)	Depth *d*: prior_B

Notes. The normalization parameter is defined as $\tilde{P}=P/P_{ref},\tilde{v}=v/v_{ref},\tilde{F}=F/F_{ref}$, $LED=\tilde{P}/\tilde{v}$, $VED=\tilde{P}$/($\tilde{v}\tilde{F}$).

**Table A2:** The feature quantity relationship among the three target models.

Target	Initial Features (*n*^raw^)	Post-Collinearity (*n*^col^)	Final Selected (*n*^opt^)	Optimal Feature Subset
Width w_1_	31	12	3	inv_VED, inv_v, inv_FSR
Height *h*	40	14	4	Fv_over_w1, F_sqLED, log_*p*, log_VED_sq
Depth *d*	30	11	3	prior_B, *p*_v_F, log_*p*

Note. Collinearity screening threshold: ∣*r*∣ > 0.92 (pairwise Pearson correlation). Values under candidate pools (*n***^raw^**) represent the columns instantiated in the script, whereas Table A1 lists the 39 unique canonical formulations defined across all categories.
